# The Conventional and Alternative Therapeutic Approaches in Arterial Stiffness Management

**DOI:** 10.3390/pharmaceutics18020166

**Published:** 2026-01-27

**Authors:** Joseph Iharinjaka Randriamboavonjy, Valeria Conti, Ines Mazza, Thierry Ragot, Charles Andrianjara, Herintsoa Rafatro, Angela Tesse

**Affiliations:** 1 Laboratory of Clinical Pharmacology Assessment, Malagasy Institute of Applied Research, Albert and Suzanne Rakoto-Ratsimamanga Foundation, Lot AVB 76, Avarabohitra Itaosy, Antananarivo 102, Madagascar; charles.andrianjara@gmail.com (C.A.); prof.rafatro@gmail.com (H.R.); 2 Department of Medicine, Surgery, and Dentistry, University of Salerno, 84081 Baronissi, Italy; vconti@unisa.it; 3 Postgraduate School of Clinical Pharmacology and Toxicology, University of Salerno, 84081 Baronissi, Italy; imazza@unisa.it; 4 UMR 9018 Aspects Métaboliques et Systémiques de L’oncogenèse pour de Nouvelles Approches Thérapeutiques (METSY), Université Paris-Saclay, CNRS, Gustave Roussy, 114 rue Edouard Vaillant, 94805 Villejuif, France; thierry.ragot@gustaveroussy.fr; 5 Laboratory of Food Cosmetic Remedy & NaturoEstheticoTherapy e-Coaching/Experting Cabinet, Gasy Products Services, Lot IIN 187 G Bis A, Analamahitsy, Antananarivo 101, Madagascar; 6 Nantes Université, Faculty of Sciences and Technologies, INSERM, The Enteric Nervous System in Gut and Brain Disorders, 44035 Nantes, France

**Keywords:** arterial stiffness, physical activities, conventional drugs, medicinal plants, mechanisms of action

## Abstract

Arterial stiffness encompasses the global structural and functional modifications that induce progressively increased vascular rigidity, whether associated with pathological cardiovascular or metabolic alterations or not. This narrative review highlights the comparative effects of physical exercise practices, current drug treatments, and approaches based on the use of medicinal plants on arterial stiffness, due to metabolic and/or blood pressure disorders. This review would provide up-to-date information for further experimental and clinical studies concerning the prevention/therapy of high glucose levels and vascular remodelling. Indeed, it is known that physical activities can reduce high blood glucose and blood pressure, allowing the prevention of arterial stiffness. Concerning conventional drugs, some are used to treat arterial stiffness, but their effectiveness is generally limited to treating components of the disease. Eighteen medicinal plant species, belonging to fourteen different botanical families, have potential activities against arterial stiffness in preclinical and/or clinical studies. Several plant extracts reduced the parameters implicated in vascular stiffness, such as the Pulse Wave Velocity, the Augmentation Index, and the Cardio–Ankle Vascular Index. Some plant extracts reduced arterial stiffness by primarily lowering glycemia and/or blood pressure in animal models, which has also been confirmed in humans. By reducing arterial stiffness, plant extracts or derived bioactive compounds not only improved vascular relaxation by enhancing Nitric Oxide production and/or antioxidant defences, but also inhibited inflammation-induced aortic remodelling and promoted elastin neo-synthesis. Polyphenols have often been identified as the main effective compounds involved in these beneficial effects. However, only a few studies explained the mechanisms associated.

## 1. Introduction

**Figure 1:**
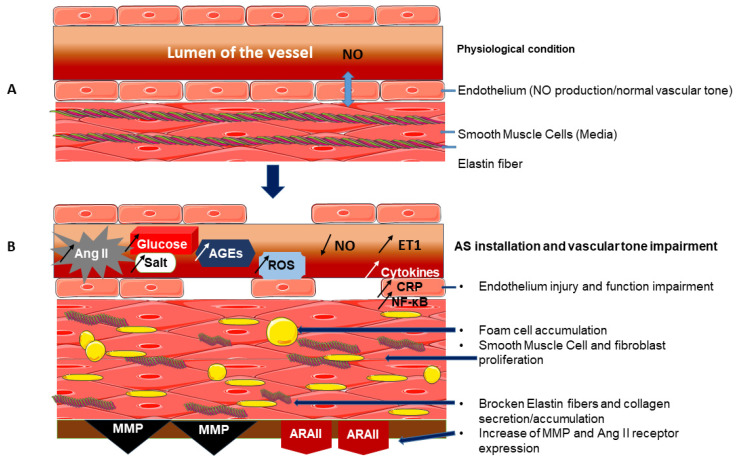
General physio-pathological mechanisms of arterial stiffness. (**A**): Healthy vessel in the physiological state. (**B**): Pathological vessel showing the mechanisms of vascular dysfunction and the development of arterial stiffness (AS). Ang II: Angiotensin II; AGEs: Advanced Glycation End-products; ROS: Reactive Oxygen Species; NO: Nitric Oxide; MMP: Matrix Metalloproteinase; ARAII: Angiotensin II receptors; CRP: C-reactive protein; NF-κB: Nuclear Factor Kappa-B. ↗ = increased; ↙ = decreased; ↓ = from to.

Arterial stiffness (AS) refers to the modifications in vascular wall composition and structure resulting from physiological changes, such as those occurring during normal vascular ageing, and/or due to pathological risk factors, including high salt/sugar consumption and hypertension [1]. Indeed, as reported by Alvim et al. [2], inflammation-associated diabetes and/or hypertension increased collagen production and diminished vascular elastin synthesis. This leads to endothelial dysfunction and vascular remodelling due to increased arterial fibrosis and hypertrophy (Figure 1).

In the context of diabetes, a review of Lacolley et al. [3] focused on the deleterious mechanisms of hyperglycaemia on vascular integrity. High circulating glucose stimulates Vascular Smooth Muscle Cell (VSMC) proliferation, increasing the production of Advanced Glycation End-products (AGEs) and vascular accumulation of collagen. Moreover, Matrix Metalloproteinase (MMP) and Angiotensin II (Ang II) receptor expression is increased in the vascular wall. Furthermore, insulin resistance, the inflammatory processes associated with the interaction between vascular and immune cells, as well as increased collagen synthesis, induce AS. Petrie et al. [4] also demonstrated the involvement of the pro-oxidant pathway. AGE mechanisms were associated with oxidative stress by stimulating Reactive Oxygen Species (ROS) production, which in turn enhanced AGE formation in the diabetic state, thus promoting the AS process. The activation of the AGE Receptors (RAGE) was reported to stimulate ROS-dependent injury and to trigger inflammatory pathways. Consequently, vascular inflammation and oxidative stress lead to AS-associated fibrosis and arterial calcification due to fibroblast secretions. AGE production is also marked by a high level of methylglyoxal (a precursor of AGEs) in diabetes. Through inflammatory reactions, this molecule could cause collagen formation and elastin degradation by ROS production, contributing to vascular stiffening [5]. According to Jia et al. [6], the impairment of insulin responses during diabetes alters vascular tone. Thus, the molecular mechanisms underlying arterial dysfunction involve Ang II binding to Angiotensin II type 1 (AT1) receptors, endothelial Nitric Oxide (NO) reduction, and oxidant-associated inflammation (Figure 1). Aroor et al. [7] described another mechanism. Blood vessel intima could be subjected to structural and functional modifications differently in conductance and small resistance arteries. Hence, Ang II inhibited insulin-induced NO production in endothelial cells and activated both Extracellular Signal-Regulated Kinases 1 and 2 (ERK1/2) and Endothelin-1 (ET-1), a potent vasoconstrictor contributing to vascular stiffness and increased vascular tone. The Renin–Angiotensin–Aldosterone System (RAAS) could inhibit insulin cellular pathways, resulting in vascular infiltration of inflammatory cells and AS development.

Hypertension is considered one of the main risk factors for death worldwide. Concerning the role of arterial hypertension in AS, Morgan et al. [8] reported that high systolic blood pressure (SBP) decreases arterial elasticity and causes large blood vessel rigidity through several modalities. For instance, ageing-associated hypertension processes occur at different levels: phenotypical alteration of VSMC, adventitia disorganisation due to elastin fragmentation, calcium deposition, and collagen fibre formation [9]. Several reviews have described the link between high blood pressure (BP) and AS: prohypertensive factors or vasoconstrictor agents, such as salt, Ang II, and ET-1, accelerate AS and MMP-mediated vascular wall collagen accumulation. Transforming Growth Factor-Beta 1/Suppressor of Mothers Against Decapentaplegic (TGF-β1/Smad) acts as an effector involved in myofibroblast activation/proliferation and monocyte/macrophage infiltration. Therefore, oxidative stress and vascular inflammation promote elastin fibre rupture, VMSC hypertrophy, and arterial fibrosis associated with AS [2,10,11,12].

High BP is often associated with other disease complications, such as diabetes, which contribute to increased cardiovascular risk. The number of people with diabetes was around 451 million in 2017 and will be 693 million in 2045 [13]. The prevalence of this metabolic disorder is increasing annually, and it is often associated with obesity, insulin resistance, nephropathy, and cardiovascular disorders, affecting 32.2% of worldwide type 2 diabetic patients. Hypertension is 1.5 to 2 times higher in these patients [14,15]. In some European Union countries, the prevalence of hypertension among type 2 diabetics is high: 89.4% in Germany, 79.4% in Spain, and 80% in Italy [16,17,18]. Concerning Madagascar, in the city of Antananarivo, 70.78% of type 2 diabetics suffer from hypertension [19]. Tian et al. [20], in a prospective AS cohort study, demonstrated the importance of AS in predicting diabetes and that AS is related to both hyperglycaemia and hypertension. Thus, preventing and/or treating AS could be beneficial in inhibiting diabetes vascular complications and in reducing hypertension-induced vascular damage. This narrative review describes numerous approaches to the prevention and/or treatment of AS. Some of them are currently applied, such as the use of drugs or sport activities. Here, we focus on in-depth research aimed at identifying natural alternative resources, mainly plant compounds used in traditional medicine for the management of cardiovascular diseases. Physical exercise and current drug treatments will be mentioned to compare the mechanisms involved in the vascular benefits of medicinal plants (improvement of endothelial function, anti-inflammatory and antioxidant effects). Phytochemicals targeting AS in preclinical and clinical research will be discussed, which could contribute to future research by providing additional scientific data useful for clinical trials in the prevention/treatment of AS.

## 2. Care Approaches and Tools to Modulate the Mechanisms of AS Development

### 2.1. Parameters Confirming AS

Before discussing the chemical or natural compound-based approaches aimed at preventing or treating the development of atherosclerosis and its vascular manifestations, this section summarises the main parameters used to assess vascular stiffness. In addition, currently available therapeutic approaches for atherosclerosis are briefly reviewed.

Echocardiographic cardiac and vascular measurements are the first proposed methods for assessing vascular stiffness. However, BP could lead to confusion in the interpretation of disease diagnosis. Thus, different methods were adopted to investigate AS. The physical and mechanical properties of blood vessels may be considered. Teixeira et al. [21] first showed the importance of the Carotid–Femoral Pulse Wave Velocity (cf-PWV) parameter in measuring vascular stiffness in humans. Then, echocardiography coupled with pulse wave Doppler allowed analysis of the local properties of the vascular wall. Clinical trials of AS in patients with type 2 diabetes, hypertension, and systemic lupus erythematosus, have confirmed an increase in cf-PWV [22,23,24]. As a pulse propagates within blood vessels, the increase in cf-PWV is reflected by a wave delay due to vascular wall rigidity. Therefore, arterial compliance and distensibility were lower in these patients, suggesting a reduction in arterial elasticity related to endothelial dysfunction and elastin disorganisation. Another point of view suggests that hemodynamic parameters, including BP, affect cf-PWV, which could also be misinterpreted in other cardiovascular diseases [25]. Consequently, Shirai et al. [26] proposed another index, the Cardio–Ankle Vascular Index (CAVI), which is independent of BP and provides an overview of all arterial rigid segments from the heart to the ankle. Furthermore, the CAVI value was high in AS associated with diabetes and hypertension.

### 2.2. Existing Approaches Used in AS Prevention and Treatment

AS is one of the early vascular ageing symptoms; indeed, ageing induces elastin loss, increased oxidative stress-associated inflammation, enhanced endothelial dysfunction, and stiffness of smooth muscle cells in the vasculature. For this purpose, DuPont et al. [27] identified different factors in AS mechanisms and attributed them to ageing, obesity, and related gender-specific molecular mechanisms. Ashor et al. [28] defined AS as the result of a degenerative process of the elastic artery extracellular matrix due to the effect of risk factors, while advanced AS is a biomarker of cardiovascular risk stratification.

High blood pressure, diabetes, metabolic syndrome and, above all, chronic kidney disease (CKD) can also exacerbate AS [29]. A review by Inserra et al. [30] discusses the mechanisms underlying the bidirectional relationship between CKD and AS. In CKD, metabolic and neurohormonal alterations, including hyperphosphataemia, hyperuricaemia, sodium retention, activation of the RAAS, and increased sympathetic activity, promote hypertension, atherogenic dyslipidaemia, and premature vascular ageing [31]. All these factors contribute to the development of endothelial dysfunction, characterised by reduced bioavailability of NO and increased oxidative stress, as well as structural remodelling of the arterial wall, with inflammation of the intima, fragmentation of elastic fibres, collagen accumulation, and calcification of the media [32]. The resulting AS leads to an increase in systolic and pulsatile pressure, with excessive pulse wave transmission (PWT) to the microcirculation. These hemodynamic alterations promote glomerular damage, renal fibrosis, and the progression of CKD, as well as the risk of cardiovascular and cerebrovascular events, creating a vicious circle between vascular dysfunction and renal failure [33]. In fact, increased AS and endothelial dysfunction activate adhesion molecules, such as Monocyte Chemoattractant Protein-1 (MCP-1), and cytokines that promote thrombotic events [34,35,36]. Dendritic cells and T-lymphocytes play an essential role in synthesising pro-atherogenic cytokines [IL-2, IL-18, and Interferon Gamma (IFNγ)] [37]. Vascular inflammation enhanced by CKD promotes vascular stiffening by stimulating fibrosis and proliferation of the VSMC [32].

Several theories have been proposed to explain the cellular basis of atherosclerosis; the most widely accepted is Ross’s hypothesis of damage response. This model describes atherosclerosis as a chronic inflammatory and proliferative response to endothelial damage or dysfunction [38]. Endothelial damage, caused by factors such as chronic hyperlipidaemia, metabolic and chemical insults (e.g., homocysteine, uremic toxins), infections, immune-mediated mechanisms, and mechanical stress, increases vascular permeability and promotes leukocyte and platelet adhesion [39]. The subsequent infiltration of plasma constituents, particularly lipoproteins, together with Platelet-Derived Growth Factors (PDGF) and cytokines, stimulates the migration and proliferation of VSMC into the media and the expansion of resident intimal smooth muscle cells. Simultaneously, the recruitment of inflammatory cells supports lipid accumulation and extracellular matrix remodelling, ultimately leading to the formation and progression of atherosclerotic plaques [40].

Therefore, can we consider AS as a degenerative disease? Would it be curable? Until now, mainly two approaches have generally been applied to prevent or treat AS. First, healthy lifestyle changes are proposed to patients to restore vascular function, in particular a regular sporting activity and a healthy anti-inflammatory and antioxidant diet with low levels of sugar and salt, to avoid metabolic alterations and the development of hypertension. Second, some chemical compounds, usually regulating pathways involved in cardiovascular diseases and metabolic syndrome, might be used to alleviate vascular stiffness, according to specific pathological factors and mechanisms of the disease.

#### 2.2.1. Physical Exercise and Other Healthy Approaches Involved in AS Prevention

- The clinical trial PULSE (Blood Pressure Utilizing Self-monitoring after Exercise) by Kiernan et al. [41], assessing post-exercise cardiovascular benefit, concluded that both hypotension and vasodilation were observed as one of post-exercise phenomenons due to hemodynamic adjustments occurring from aerobic or exercise recovery, which should be controlled to avoid post-exercise cardiovascular instability. Indeed, a clinical study by Liu et al. [42], conducted on 70 prehypertensive patients, demonstrated the crucial role played by the central baroreflex pathway in decreasing BP, inducing the phenomenon called post-exercise hypotension. Brandão Rondon et al. [43] enrolled 24 elderly hypertensive patients and confirmed this observation, suggesting that post-exercise hypotension is due to peripheral vascular resistance reduction. According to Santos et al. [44], a crossover trial conducted on 20 patients with resistant hypertension supported these findings.
- According to a randomised clinical trial conducted in 16 type 2 diabetic patients, Myette-Côté et al. [45] demonstrated that a low-carbohydrate diet induced a reduction in blood glucose levels and mobilised peripheral blood monocytes. This glucose-lowering effect was more beneficial when combined with post-meal walking. In agreement, Chiang et al. [46] studied the effects of moderate-intensity exercise on blood glucose response in 66 type 2 diabetes patients through a prospective longitudinal evaluation. In this clinical trial, blood glucose progressively declined and stabilised over twelve weeks of training. Moreover, exercise in the afternoon or evening had increased ability to induce lower glucose levels compared to those found after morning exercise. Both prospective observational studies, conducted on 197 and on 100 pregnant women, respectively, showed that even maintaining moderate and regular physical activity, as well as applying long-term resistance exercise, would be recommended for pregnant women displaying gestational diabetes [47,48]. Motahari-Tabari et al. [49] conducted a randomised clinical study in 55 type 2 diabetic women, which promoted low plasma glucose due to medical treatment combined with aerobic exercise to target insulin resistance.
- Fernberg et al. [50] assessed the relationship between sedentary behaviour and cardiovascular disease in 658 young healthy and non-smoking adults, participating in the LBA (cross-sectional Lifestyle, Biomarkers and Atherosclerosis) trial. A progressive decrease in AS was observed among people practising a moderate or vigorous physical activity. Park et al. [51], in a randomised clinical trial involving 72 patients with peripheral artery disease, suggested that an aquatic walk exercise could be an effective therapy to reduce AS, improving heart rate, cardiorespiratory capacity, and strengthening muscles and physical function. According to Park et al. [52], in a clinical pilot study involving 20 patients, a combination of aerobic and resistance exercise effectively enhanced the quality of life in obese older adults with concomitant AS reduction. This observation was confirmed by Endes et al. [53] following the SAPALDIA 3 Cohort study in 1908 elderly people, which showed low AS after vigorous physical activities. Interestingly, a Maastricht Study by Vandercappellen et al. [54], conducted in 1699 patients, proposed that higher-intensity physical activity might be a main strategy to reduce cardiovascular disease risk, such as AS, particularly in type 2 diabetes subjects. The Stamatelopoulos et al. [55] cross-sectional study, involving 625 healthy subjects, concluded that PWV reduction occurred in normal-weight postmenopausal women after physical activity.
- Physical exercise restores cardiovascular function. Benefits include parasympathetic nervous system stimulation and the proper use of glucose, which might prevent AS development associated to diabetes and hypertension.
- Other lifestyle changes, as well as physical activity, could have beneficial effects. For instance, Takami and Saito’s [56] observational study, conducted in 70 subjects who stopped smoking, suggested an essential age-related reduction in vascular stiffness. Moreover, a diet therapy containing omega-3 (ω-3) supplementation reduced PWV and CAVI in metabolic syndrome and hypertensive patients, enhancing arterial distensibility. As reported by Sacks et al. [57] in DASH (Dietary Approaches to Stop Hypertension), a randomised trial conducted in 412 participants, the diet was effective in reducing AS. This non-pharmacological approach was mainly able to decrease oxidative stress, preventing vascular free radical deleterious effects. This finding was confirmed by Imamura et al. [58] in a randomised controlled study involving 50 type 2 diabetic patients, demonstrating that resveratrol treatment decreased CAVI and SBP without significant changes in metabolic parameters.

#### 2.2.2. Antihypertensive Drugs in AS Treatment

Conventional drugs mainly acting in the circulatory system also manage AS-related high BP in different ways:

- Vascular muscle modulation was evidenced in 25 healthy subjects and 25 arteriosclerotic patients [59]. Vascular relaxation by nitroglycerin reduced CAVI and repaired AS injuries in muscular arteries.
- System or receptor blocking/inhibiting: RAAS inhibitors were the most effective in AS treatment, probably by regulating vascular wall fibrosis formation. Targeting arterial structure is the most successful AS therapy. Angiotensin-Converting Enzyme inhibitors (ACEi) and Angiotensin Receptor Blockers (ARBs) reduced AS in patients with resistant hypertension without improving arterial compliance. Furthermore, a prospective clinical study, conducted by Palić et al. [60] on 31 hypertensive patients, proved AS reduction after zofenopril treatment. As demonstrated by Jung et al. [61], in a study where telmisartan was administered to 39 patients with essential hypertension, the treatment decreased Brachial–Ankle Pulse Wave Velocity (ba-PWV) and increased Flow-Mediated Dilation (FMD). Mahmud and Feely [62] described the effects of β-adrenergic antagonists, or Beta-Blockers (BBs), in 40 hypertensive patients, demonstrating that atenolol and nebivolol diminished BP and PWV associated with NO production and vasodilation. According to a trial by Sasaki et al. [63], involving 40 type 2 diabetic patients affected by hypertension and nephropathy, Calcium Channel Blockers (CCBs), such as efonidipine, decreased CAVI with a reduction in circulating aldosterone and the oxidative stress marker 8-hydroxy-2′-deoxyguanosine. As shown by Wang et al. [64], in a randomised double-blind clinical trial conducted in 269 hypertensive patients, a twenty-week treatment with amlodipine and lacidipine decreased ba-PWV and reduced AS. These classes of drugs are used especially for vascular contraction inhibition and must be combined with other antihypertensive compounds to overcome AS.
- Direct Renin Inhibitors (DRI): Virdis et al. [65], in a three-month study involving 50 patients with essential hypertension, showed that aliskiren decreased BP, central Pulse Pressure (PP), Augmentation Index (AIx), and aortic PWV. In addition, in 24 type 1 diabetic patients, Cherney et al. [66] reported that aliskiren enhanced endothelial function by increasing the FMD. These effects reduce AS in both diabetic and hypertensive patients.

#### 2.2.3. Antidiabetic Drugs Reducing AS

Conventional drugs against metabolic diseases might reduce AS-related diabetes symptoms/complications. However, certain parameters reflecting AS should be taken into account. The prospective international study by Toupochian et al. [67] conducted in 1664 and 560 patients with and without metabolic syndrome, respectively, showed that PWV was increased in patients with metabolic disorders, whereas CAVI was associated only with blood pressure and glycaemia, and its value decreased inversely as the waist circumference (WC). As CAVI covers a wider aortic region than PWV, sensing the descending aorta, wave propagation could be slowed down by the enlarged body volume caused by high WC. Thus, consideration should be given to set appropriate parameters for analysing AS-associated metabolic syndrome, such as vascular dysfunction and stiffness. Therefore, drugs implicated in diabetes and/or obesity treatments might help in selecting AS therapeutic management.

There are several studies using drugs to control glycemia in the context of AS, exhibiting some beneficial effects in restoring vascular structure and function:

- Regarding sulfonylureas: a randomised clinical study by Nagayama et al. [68], using glimepiride administered to 40 type 2 diabetic patients, demonstrated that a six-month treatment reduced CAVI and lipoprotein lipase (an insulin resistance indicator), as well as the oxidative stress marker, 8-hydroxy-2′-deoxyguanosine.
- Concerning biguanides, metformin (MF) decreased PWV in a study by Bjornstad et al. [69], conducted in 48 adolescent type 1 diabetic patients. Furthermore, a study conducted with MF in 210 type 2 diabetic patients by Chen et al. [70] showed a reduction in inflammatory molecules, such as cytokines.
- Sodium-Glucose Cotransporter 2 (SGLT-2) inhibitors, called gliflozins, were known to improve FMD, while Glucagon-Like Peptide 1 Receptor Agonists (GLP-1 RAs) and Dipeptidyl Peptidase 4 (DPP-4) inhibitors decreased PWV. Two clinical studies, involving 60 and 40 type 2 diabetic patients, respectively, demonstrated AS reduction in both groups [71,72]. As shown in clinical trials by Bosch et al. [73] in 58 type 2 diabetic patients and by Cherney et al. [74] in 42 type 1 diabetic patients, empagliflozin reduced SBP and pulse pressure-associated AS related to high-sensitive inflammatory marker C-reactive protein (hsCRP) reduction. The susceptible mechanisms of these beneficial effects were proposed by Neutel et al. [75] and Soares et al. [76] in aged mice models, showing the reduction of collagen type I and TGF-β protein expression in the media of the infrarenal aorta of aged mice. At the mesenteric artery level, the treatment enhanced endothelial function, characterised by the phospho-eNOS/eNOS ratio increase and by the reduction of the oxidative stress marker, Malondialdehyde (MDA).
- In a similar way, a GPL-1 RA, liraglutide, used by Lambadiari et al. [71] in a clinical trial, reduced cf-PWV and increased FMD, indicating AS inhibition. These effects were associated with MDA reduction, suggesting an antioxidative property of this drug in humans. Experimental studies, using diabetes induced by streptozotocin (STZ) in rats and in cultured human umbilical vein endothelial cells (HUVECs), demonstrated that liraglutide inhibited Nicotinamide Adenine Dinucleotide Phosphate (NADPH) oxidase, or NOX, via Protein Kinase A (PKA) activation. This finding was confirmed by the inhibition of both gp91^phox^ and p22^phox^ in endothelial cells. Liraglutide also showed an anti-inflammatory effect by reducing Nuclear Factor Kappa-B (NF-κB) protein expression/activation in the presence of Tumour Necrosis Factor-Alpha (TNF-α) [77,78].
- DPP4 inhibitors (DPP4-i), also called gliptins, have effects on endothelial cell homeostasis and proliferation. As reported by Stampouloglou et al. [79], in a clinical trial conducted in 118 diabetic patients, DPP4-i decreased PWV in addition to weight loss. In HUVECs exposed to hydrogen peroxide (H_2_O_2_) and high glucose, anagliptin increased cell viability and inhibited cell senescence by reducing the release of interleukins and pro-oxidant molecules, such as interleukin 1-β (IL-1β) and lactate dehydrogenase (LDH) [80]. In an in vivo study using the Aortic-Banded Mini Swine model, saxagliptin-dependent vascular stiffness reduction was due to the decline of NF-κB-induced inflammation, AGEs, and nitrotyrosine in coronary arteries [81].

#### 2.2.4. Cholesterol-Lowering Drugs and AS

In the last decade, the use of statins has provided beneficial effects in reducing metabolic syndrome-associated cardiovascular complications. Indeed, these drugs decreased PWV by interacting with the sympathetic nervous system and pro-oxidant factors impacting vascular endothelial function [82,83]. As reported by Alidadi et al. [84] and Gepner et al. [85], simvastatin modulated vascular elasticity in inflammation-induced AS. An in vitro study conducted by Lampi et al. [86] elucidated the simvastatin mode of action. In bovine aortic endothelial cells, the treatment decreased Ras Homolog family member A (RhoA) activity and enhanced Ras-related C3 botulinum toxin substrate 1 (Rac1) protein expression in endothelium. This led to a reduction in vascular contractility, ameliorating vascular tone and cellular matrix organisation modified by AS. Atorvastatin was also effective against hypertension and metabolic syndrome-induced AS by reducing central aortic pressure and PWV, with a decrease in vascular inflammation markers, including CRP and OsteoProteGerin (OPG) [87,88]. Clinical observation was conducted by Wang et al. [89] using atorvastatin in elderly hypertensive patients. This statin demonstrated an antioxidant mechanism during a six-month treatment. This was correlated with AS reduction and copper⁄zinc-containing Superoxide Dismutase (Cu⁄Zn-SOD) activity increase. Statins also acted as antidiabetic drugs, and their effects proved their efficacy against the deleterious effects of inflammation-associated oxidative stress, thus suggesting their potential ability to reduce AS. Another study by Saiki et al. [90] evaluated the effects of pitavastatin compared with atorvastatin in hypercholesterolemic patients at risk of death caused by heart or blood vessel diseases, myocardial infarction, and stroke. One-year chronic treatment reduced CAVI and the risk of developing heart disease in treated patients compared with atorvastatin. Since CAVI reflects AS, taking pitavastatin may prevent AS development, which was not measured in this study. In other words, the improvement that was achieved with atorvastatin may not be solely attributed to the vascular component, as other effects were obtained with the treatment.

#### 2.2.5. Probable Drug Combinations Used to Treat AS

Based on these data, antidiabetic drugs exert anti-inflammatory and antioxidant effects to reduce AS and AS-associated risk factors. However, the combination approach also exhibited beneficial effects. As suggested by Vlachopoulos [91] and Chen et al. [92], de-stiffening therapy was more effective when combined with CCBs and ACEi. These effects complemented each other by inhibiting Ang II production and preventing calcium-induced artery contraction. This treatment combination reduced collagen accumulation and vascular thickness.

### 2.3. Drug Molecular Structure-Activity Relationship

Among drugs reducing AS, the biological activity is related to the chemical structure. Thus, Fujino et al. [93] studied the effects of irbesartan and losartan on endothelial cells isolated from human coronary arteries. The antihypertensive properties of these molecules were attributed to the presence of hydroxyl and carboxyl groups, which are involved in blocking Ang II vascular effects. Therefore, they could have a similar effect on lowering AS. Concerning ACEi, Zheng et al. [94] reported that the carboxyl (enalapril) or phosphonate (fosinopril) groups interacted with the ACE C-domain, leading to inhibition of the conversion of Angiotensin I (Ang I) to Ang II.

According to the chemical study carried out by Guerrero et al. [95] and the phytochemical analysis of Hussain et al. [96], plant bioactive compounds such as flavonoids, luteolin, and quercetin are well-known to have vasoprotective properties and also possess effects against ACEi.

As reported by Labrid et al. [97], beta-blocker drugs share an oxypropanolamine structure, but changing radicals to phenol groups might affect beta-adrenergic receptor activity. In the case of nevibolol, according to Ignarro et al. [98], cardiovascular properties of this molecule were attributed to its two enantiomers (D-nevibolol and L-nevibolol), which worked in synergy to induce vascular relaxation and heart rate reduction. These effects suggest that the treatment with nevibolol might reduce cardiac afterload and vascular wall rigidity. Another class of antihypertensive drugs used in AS concerns CCBs. Masson et al. [99] demonstrated the effectiveness of amlodipine vasodilators. Indeed, the presence of charged aminoethoxy linked with dihydropyridine increased the membrane affinity of this drug and enhanced its cellular concentration in VSMC. By the same mechanism, amlodipine might act by decreasing calcium ions associated with vascular wall rigidity during AS.

Renin inhibition represents a beneficial effect in AS and hypertension management. Brown [100] highlighted the importance of its utilisation. In particular, aliskiren possesses a piperidine core with a high affinity for the renin site, binding to the enzyme substrate receptor to exert its effect, thus reducing BP and enhancing vasodilation for an extended period.

Concerning antidiabetic drugs, their efficacy against AS has also been demonstrated by certain therapeutic classes, called biguanides, according to Rusanov et al. [101]. MF chemical structure is represented mainly by two guanidine groups related to its hypoglycaemic activity. They might be involved in NO production associated with enhanced vascular relaxation in AS treatment. Furthermore, according to a review by Lymperopoulos et al. [102], SGLT-2 inhibitors, or gliflozins, are D-glucosides possessing a glucose core linked to two phenyl groups that form the aglycone part. Mechanisms include the blockage of renal glucose reabsorption in the renal proximal convoluted tube, which promotes sodium ion excretion, thus leading to BP reduction. According to Faillie [103], the first SGLT-2 inhibitor was an isolated flavonoid (glycoside phlorizin) from apple tree bark. As the cardiovascular effects of flavonoids are well-known, glycoside phlorizin might be able to exert artery de-stiffening activity, similar to other flavonoid bioactive compounds.

Another molecular mechanism triggering insulin secretion was attributed to GLP-1 RAs. For this purpose, Vilsbøll [104] characterised the chemical structure of liraglutide, a GLP-1 RA, in comparison with the natural agonist, GLP-1. This analogue is made up of thirty amino acids with lysine at position 34 instead of arginine, and its binding to receptors reproduces the physiological effect of GLP-1. In the context of AS, controlling the body glucose use via autocrine pathways might facilitate the protection of VSMC and endothelium in diabetes-associated hypertension.

Concerning DPP4-i, Mathur et al. [105] elucidated their mechanisms. Indeed, as a serine protease, DPP4 degrades incretin hormones involved in stimulating insulin production. Thus, DDP4-i increase β-cell function in the management of glucose utilisation and might lead to cardiovascular protection, as mentioned above [79,80,81]. Moreover, Guedes et al. [106] reported that DPP4-i presented various chemical forms and were classified according to their effect as mimetics of the DPP4 enzyme. Even in the same class, gliptins possess various rings, such as a xanthine base for linagliptin and adamantane for saxagliptin, that confer their antidiabetic properties and potentially contribute to the reduction of AS.

As dyslipidaemia contributes to cardiovascular dysfunction, the use of statins, considered amphiphilic compounds, provided beneficial effects in AS. Murphy et al. [107] described the cell signalling pathways of statins. The presence of a 3-Hydroxy-3-Methyl-Glutaryl-Coenzyme A (HMG-CoA) functional group confers activity against HMG-CoA reductase, responsible for hepatic cholesterol synthesis. In the case of simvastatin, the pharmacophore is the dihydroxyheptanoic acid, which, by reducing serum cholesterol, prevents arterial diseases and vascular stiffness [107].

### 2.4. Plants Used in Vascular Stiffness Alleviation

In line with the aim of this review, the molecular similarities of some natural molecules with drugs currently used to prevent and/or treat AS have allowed us to identify, in the literature, eighteen plants, often used in traditional medicine, belonging to fourteen different botanical families with potentially interesting effects against AS. Several studies were conducted using extracts or bioactive compounds from these selected plants in animal models and in clinical trials. In most of the works cited below, materials harvested from plants were used directly in powder form, or they were first extracted, and aqueous or a hydroalcoholic extract was administered to animals and/or humans. Certain fractions or bioactive compounds were isolated by some authors to elucidate their biological effects and action mechanisms.

#### 2.4.1. Methodology

For selected plants, in vitro studies were also included: ACE inhibition, glycation-induced collagen formation in neutrophil cells, VSMC from 24-month-old mice, as well as H_2_O_2_-induced senescence in endothelial cells. In order to highlight plant, plant extract or isolated plant compound activity against AS, several studies used various animal models: ageing- or postmenopausal-induced AS in aged C57BL/6 mice/rats, ovariectomized female Wistar rats, ovarian polycystic syndrome rats, alloxan-, STZ-, or KK-Ay/T-induced diabetic mice/rats, obese mice or High Fat and Fructose Diet (HFFD)-treated rats, L-NG-Nitroarginine Methyl Ester (L-NAME)-induced hypertensive Wistar rats or Spontaneously Hypertensive Rats (SHR), and cadmium or high-salt (sodium chloride or NaCl) diet (HSD)-induced aortic damage with hypertension and oxidative stress in rats. These different models were chosen to demonstrate probable physiological or pathological origins of factors inducing AS. Nevertheless, phenotypically, these models present, in general, differences in AS dynamic or structural aspects, which sometimes limit the interpretation of the results. A synthesis of the cell culture and animal models used in the preclinical studies conducted with each of the eighteen plants studied in this review is shown in Table 1.

Some of the plants, plant extracts, or isolated compounds discussed in this review were also tested in clinical trials. In order to assess the influence of treatment on AS parameters, some of these plants were only tested in clinical studies conducted on healthy young subjects. The use of healthy subjects is not always appropriate to evidence the anti-inflammatory, antioxidant, and metabolic effects associated with AS development. Other clinical trials were conducted by administering the natural treatments to patients with different pathologies characterised by AS development, especially patients with cardiovascular or metabolic diseases, as well as for pathological and healthy ageing. For this purpose, some studies were conducted on elderly humans or postmenopausal women developing AS. Different human cardiovascular pathologies were also the aim of other clinical trials in which the plants were administered to hypertensive subjects or patients with stable angina and coronary artery stenosis. Other clinical studies also focused on the effects of the plants or plant extracts in metabolic pathologies, such as type 2 diabetic patients developing hypertension or obese/metabolic syndrome patients.

It is important to note that the gender difference in the natural treatment effects was not always considered in the clinical studies cited in this review, limiting the comprehension of their possible gender-dependent biological effects. Some of these trials were conducted with few enrolled subjects (9–25 healthy subjects or patients) and can be considered pilot studies that require further investigation to confirm the preliminary findings. A synthesis of the pathology or health status of the enrolled subjects, the number of subjects, and their gender for the clinical studies conducted with several plants is presented in Table 2.

#### 2.4.2. The Plants of the Araliaceae Family

Among the plants belonging to the Araliaceae family, we focused our attention on three species. The first s ispecies *Acanthopanax senticosus* (Rupr. and Maxim) Harms, or *Eleutherococcus senticosus* (Rupr. & Maxim.), also known as Ciwujia, Gasiogapi, or Siberian Ginseng. This plant is used by Chinese and Russian populations to improve the physiological functions of liver and kidneys. In Chinese traditional medicine, Ciwujia was used to alleviate cerebral arteriosclerosis, thrombosis, embolism, and coronary heart disease [108].

In the context of AS, we have not found preclinical studies concerning the evaluation of the effect of Gasiogapi fruit extract on an animal model of vascular stiffness. Nevertheless, as diabetes and obesity might involve AS onset, Saito et al. [109] suggested that a 12-week treatment with the Siberian Ginseng fruit extract, at doses of 500 and 1000 mg/kg, reduced glycaemia (* *p* < 0.05), liver triglycerides (* *p* < 0.05), and total cholesterol (TC) (* *p* < 0.05). The mechanisms included an increase in hepatic phospho-AMPK (pAMPK) (* *p* < 0.05) and in cytochrome P450 7A1(CYP7a1) (* *p* < 0.05) gene expression with the treatment of high-fat diet (HFD)-induced obesity in mice. The presence of lignans possessing two phenylpropane cores, such as Eleutheroside B (syringin), or flavonoids, such as quercetin, could confer reduced AS activity to Ciwujia. This hypothesis is supported by the study by Huang et al. [108], characterising the hypoglycaemic, antihyperglycaemic, and antioxidant properties of these bioactive compounds.

According to a 12-week randomised clinical study conducted by Oh et al. [110] in 76 men who were cigarette smokers with hypertension and metabolic disorders, the treatment of patients with aqueous fruit extract (500 or 1000 mg/day) reduced vascular stiffness by decreasing both ba-PWV and BP values in comparison to the placebo group. The lowest dose was more effective for ba-PWV (** *p* = 0.007) and SBP (* *p* = 0.044) reduction, without a significant effect on diastolic blood pressure (DBP) or FMD, indicating that the treatment effects were not dose-dependent. The increase in vasodilation was linked to an improvement in endothelial function and a larger phospho-eNOS/eNOS ratio, but there was no significant impact on oxLDL plasma levels.

Ciwujia fruit water extract particularly decreases both AS parameters and BP, which represents a potential advantage in hypertension-associated AS management. The increase in NO production may contribute to stabilising vascular tone and BP. In agreement, by restoring arterial function, Gasiogapi fruit extract enhanced vascular endothelial function, which is a dynamic component of AS. By considering preclinical blood glucose, TG, and cholesterol lowering in obese mice, during the same period of treatment, the tendency to decrease metabolic parameters in humans could confirm the vascular protective effect of this plant extract. Attenuating hyperglycaemia or metabolic disorder-induced deleterious vascular effects could contribute to reduce diabetes-associated vascular oxidative stress, allowing arterial tone restoration. As the vascular de-stiffening effect of the Siberian Ginseng fruit aqueous extract is associated with the activation of the NO pathway and blood glucose lowering, it might protect vascular endothelium against high glucose-induced blood vessel inflammation and wall thickness by restoring vascular wall elasticity via the improvement of the NOS pathway and/or control of glucose utilisation by pancreatic β-cells releasing insulin.

Another plant of the Araliaceae family with potential properties against AS is *Panax ginseng* C.A. Meyer, also called Ginseng, Korean Red Ginseng (KRG), or Korean White Ginseng (KWG), which is widely used in traditional Asian medicine. As the genus name “Panax”, meaning panacea, reflects the effectiveness of this plant against any disease, the Ginseng roots/rhizomes have specific cardioprotective effects and are currently used to treat blood vessel dysfunction in traditional Chinese and Ayurvedic medicines [111]. Pharmacological studies concerning its vasodilation activity represent relevant interest in controlling arterial function. Choi et al. [112] tested in aged mice the effect of 10 and 20 mg/day of KRG root water extract for 4 weeks. The main findings of this study were arginase activity inhibition (* *p* < 0.05), with subsequent enhanced eNOS activity/NO production (* *p* < 0.05/** *p* < 0.01), and the improvement of acetylcholine (Ach)-dependent aorta relaxation ** *p* < 0.01) in treated animals. Moreover, KWG extract inhibited lipid peroxidation (** *p* < 0.01) and reduced ROS vascular formation (** *p* < 0.01) and peroxynitrite plasma concentration (** *p* < 0.01). These results proved the KRG involvement in restored vascular function in ageing animal models. Its use was beneficial in arterial elasticity repair and in decreasing the ageing-dependent vascular rigidity. According to Zhang et al. [113], in a study conducted in STZ-induced diabetic mice, 8 weeks of administration of 10, 20, 40, and 60 mg/kg/day of ginsenoside Rb1, the major active component of KRG, increased Ach-induced aorta relaxation (* *p* < 0.05) and decreased the degradation of elastic modulus (Ep) (* *p* < 0.05) and arterial stiffness index (β) (* *p* < 0.05), indicators of AS reduction. The maximal effects were observed at the dose of 40 mg/kg. The vascular mode of action included the inhibition of collagen I/III aorta expression (* *p* < 0.05) and oxidative stress diminution. Rb1 upregulated phosphorylated AMPK (* *p* < 0.05) and decreased TGF-ß1/Smad2/3, MMP-2, and MMP-9 expression (* *p* < 0.05) in high glucose-treated VSMC. El-Khayat et al. [114] investigated the antidiabetic and antioxidant effects of KRG (22.5 mg/day) in STZ-induced diabetic rats treated for 45 days. In this model, Ginseng root extracts decreased glycemia, cholesterolaemia, triglyceridaemia, serum proinflammatory cytokines (IL–6 and TNF–α) (* *p* < 0.05), and increased plasma insulin (* *p* < 0.05) in diabetic rats. Shao et al. [115] reviewed the following ginsenoside antihyperglycemic mechanisms: inhibition of hepatic glucose production by activating AMPK pathways and improvement of glucose uptake in skeletal muscle and adipocytes via the insulin receptor substrate-1/phosphoinositide 3-kinase/protein kinase B (IRS-1/PI_3_K/PKB) pathway. Antioxidant effects were characterised by MDA enzyme decrease and an increase in SOD and GSH. As ginsenosides are the major components of Ginseng, their biological activity is based on their chemical structure, which consists of four steroid cores and thirty carbon atoms. Sugar position mainly differs among ginsenosides, but their reported biological activities are similar among these bioactive compounds: antihypertensive, anti-ageing, anti-inflammatory, and antioxidant properties [116]. Inflammation and oxidative stress-associated hyperglycaemia are involved in diabetes-induced AS. Thus, KRG inhibition of both cytokines and glucose metabolism pathways could be promising for AS management in diabetic patients.

Jovanovski et al. [117] studied the vascular effects of KRG roots in a randomised, controlled, double-blind, crossover trial evaluating plant powder administered to 17 healthy humans using different doses and modes of preparation, such as 3 g dried crushed roots, 1.22 g ginsenoside extract, and 0.21 g polysaccharides extract. The treatment with root powder and ginsenoside fraction lowered AIx (* *p* = 0.03) without affecting BP. In the case of type 2 diabetes, an acute, randomised, placebo-controlled, double-blind, crossover trial using three doses (1, 3, and 6 g of dried, crushed KWG roots), conducted by Shishtar et al. [118], demonstrated that the 3 g dose significantly decreased AI after 4 h of treatment compared to the control group (* *p* = 0.035) without altering BP and glycaemia (*p* = 0.29). Furthermore, Chung et al. [119] worked on 20 men with stable angina and coronary artery stenosis in a randomised, double-blind, placebo-controlled, crossover trial. A 10-week KRG treatment with 900 mg three times a day reduced AS by decreasing PWV and SBP (* *p* < 0.05). The mechanism of action suggested the reduction in Rho-Kinases (ROCK) activity (*p* = 0.068) in the treated group. In particular, Ginseng targets the dynamic and structural components of AS via NO-enhanced vascular relaxation, improving elastin structure under hyperglycaemic conditions. Targeting oxidative stress and inflammation is not only effective in diabetes-induced AS but could also contribute to limit complications in arterial hypertension. Moreover, KRG-decreased TGF-β and MMP signalling restricted VSMC growth and vascular wall thickness. Consequently, arterial hypertrophy, aortic fibrosis, and vasoconstriction associated with AS could be prevented by KRG root powder or Ginseng-isolated bioactive compounds such as ginsenoside Rb1. Apart from diabetes, KRG or KWG are effective in numerous forms of AS, but the treatment does not affect BP. Thus, their cardiovascular properties might be limited in metabolic syndrome disorders without hypertension complications.

The last plant that is part of the Araliaceae family is *Panax quinquefolius* L., commonly known as American Ginseng. This plant is used by 14.1% of the adult American population for disease prevention and treatment. Thus, it is the fifth most used plant in traditional medicine in the USA [120].

A preclinical study by Murias et al. [121] used lyophilised North American Ginseng aqueous extract as drinking water, containing a dose of 250 mg/kg/day for 12 weeks, in STZ-induced type 1 diabetic and sedentary rats receiving or not receiving this beverage and/or rats practising forced running exercise on a motor-driven treadmill. Ginseng extract only, but not exercise training alone, restored ACh-induced femoral relaxation (* *p* < 0.05) in the treated rats compared to the other groups, without affecting the carotid artery or the aorta. These data suggest a protective effect of the plant extract against the vascular detrimental effects of metabolic disorders induced by diabetes and sedentary conditions, which were reversed mainly in the femoral artery by the treatment. In another diabetic preclinical model using Sprague–Dawley (SD) STZ-injected rats, 300 mg/kg/day of the aqueous extract for 8 days induced blood glucose reduction and serum insulin increase to values comparable to those of the control group (* *p* < 0.05). Plasma NO concentration was also restored, with an increase in renal catalase and MDA reduction in the treated group [122]. Liu et al. [123] studied isolated bioactive compounds from American Ginseng in STZ-induced diabetes in mice. Malonyl ginsenosides reduced blood glucose, TG, and cholesterol, and improved insulin resistance. Thus, the cellular mechanisms are similar to those cited by Shao et al. [115], but with more detail, such as increased glucose uptake via GLUT4 and activation of both IRS-1/PI_3_K/PKB and AMPK/Acetyl-CoA carboxylase pathways.

Concerning clinical studies, a 12-week double-blind, placebo-controlled, parallel-design trial conducted by Mucalo et al. [120], evaluated an ethanolic plant root extract administered as three times two capsules of 500 mg/day in 64 patients with essential hypertension and type 2 diabetes. The results of this study evidenced that the treatment significantly decreased AI (* *p* = 0.041) and SBP (*** *p* < 0.001), indicating AS reduction in the treated patients. The vasoprotective activity of North American Ginseng was related to its antihypertensive effect, which is associated with improved vasodilation. In the case of diabetes, this plant extract reduced NO in the plasma and increased insulin. By taking these results into account, the plant extract or bioactive compounds from American Ginseng could protect the blood vessels against hyperglycaemia and hypertension by controlling glucose use and signalling pathways that contribute to reducing pro-hypertensive factors or increasing antihypertensive transduction enzymes such as eNOS and associated NO production. The use of this plant extract might prevent diabetes and high BP cardiovascular complications. The cardiovascular and metabolic protective effects of plants of the Araliaceae family, with their potential role in the prevention and/or reduction of AS development, are summarised in Table 3.

#### 2.4.3. The Plants of the Apiaceae Family

*Anethum graveolens* L., also named Dill, is part of the Apiaceae family. As reported by Meena et al. [124], Dill and Fennel (*Foeniculum vulgare* Mill.) seed powder (in equal proportions) were used by the Indian population twice daily (two spoons per dose) to control BP. At our present level of knowledge, no information concerning the activity of Fennel seeds against AS has been reported. Dill seeds aqueous extract biological activity was evaluated by Fhayli et al. [125] in an aged C57BL6/J mouse model for 3 months at doses of 5 and 10%. The treatment with 5% plant extract decreased SBP and diastolic blood pressure (DBP) (* *p* < 0.05), while the dose of 10% protected against AS by increasing the elastic modulus (Einc) (* *p* < 0.05). Moreover, the plant seed extract increased aortic distensibility associated with the amelioration of endothelial function. The mechanisms of action include elastic fibre neo-synthesis by an increase in Tropoelastin (TE) and Lysyl-Oxidase-Like 1 (LOXL-1) gene expression in the aorta and VSMC. Mishra’s study [126] in alloxan-induced diabetic mice demonstrated that the seed aqueous extract decreased blood glucose. Polyphenols are present in Dill seed aqueous extract, and these compounds are well-known to reduce vascular elastin degradation by inhibiting enzyme activity (serine proteinase, cysteine proteinase, etc.) and by preventing arterial ageing via alkaline phosphatase inhibition [126]. Thus, Dill seed extract, by reducing AS, could be associated with polyphenols contained in the extract, such as tannins and/or flavonoids. Furthermore, Dill aqueous seed extract restored both arterial structure and function during vascular ageing in mice, and these effects were associated with BP decrease and the improvement of elastin activity/expression. The treatment reduced hyperglycaemia in diabetic mice and, by controlling blood glucose, might protect blood vessels from senescence associated with AS. As this plant action focuses on the structural components of AS, the use of Dill seed aqueous extract may contribute to alleviating vascular wall remodelling. The regulation of arterial elasticity was also related to vasodilation amelioration; enhancing elastin gene expression could avoid AS progression despite ageing or diabetes and/or hypertension, thus Dill seed aqueous extract might be used both to prevent and treat AS.

*Centella asiatica* (L.) Urb., known as Spadeleaf, is another interesting plant of the Apiaceae family, involved in maintaining vascular function. In China and India, apart from skin disorders, this plant was used traditionally in cases of kidney troubles and may act as a diuretic [127].

In animal models, Hasimun et al. [128] evaluated Spadeleaf leaf ethanolic extract mixed with *Curcuma longa* rhizome in high-fat-high-fructose (HFFD)-induced hypertensive rats for a period of 28 days. In this study, at doses of 50 and 100 mg/kg/day, the extract reduced SBP, DBP, and PWV (* *p* < 0.05). At the end of the protocol, plasma NO concentration increased, suggesting enhanced endothelial vascular relaxation in this model. In another study conducted by Bunaim et al. [129], using *Centella asiatica* leaf ethanolic extract (500 mg/kg/day for 8 weeks) in L-NAME-induced hypertensive SD rats, the treatment reduced SBP, increased serum NO bioavailability, and decreased aortic MDA enzyme (* *p* < 0.05), indicating an antioxidant effect of the extract. According to Yani Mulyani et al. [130], in L-NAME-induced hypertensive Wistar rats, 28 days of treatment with a 5% Nori preparation (combination of *Centella asiatica* + *Curcuma longa*) increased aortic eNOS gene expression and decreased both ACE and inducible Nitric Oxide Synthase (iNOS) enzymes (* *p* < 0.05). Moreover, apart from the antihypertensive effect of *Centella asiatica* in rats, Kabir et al. [131] assessed the acute and chronic (28 days) antihyperglycemic activity of leaf ethanolic plant extract on type 2 diabetic rats. The treatment reduced serum glucose, TG, Low-Density Lipoprotein Cholesterol (LDL-C), and increased High-Density Lipoprotein (HDL) (* *p* < 0.05) without affecting insulin secretion. The mechanisms were characterised by inhibition of intestinal glucose absorption through reduction of disaccharidase and α-amylase enzymes and binding of glucose by bioactive compounds from whole-plant extract, leading to its incapacity to bind with the transporter [131]. This was associated with low circulating glucose levels in treated animals. Flavonoids are mainly present in Spadeleaf extract, and their anti-ACE enzyme property conferred a role in RAAS inhibition with beneficial effects in AS treatment [130]. According to these data, Spadeleaf leaf ethanolic extract acted mainly by reducing BP and oxidative stress and by enhancing endothelial function in hypertensive rat models. The implications of ROS and inflammation are well known in diabetes-induced AS and arterial dysfunction. By controlling the lipid profile in the case of type 2 diabetes, this extract might act by interacting with RAAS to regulate Ang II cellular signalling and glucose filtration by the renal system. Thus, the biological activity of *Centella asiatica* extract could help in controlling metabolic disorders associated with cardiovascular diseases in sedentary human patients. Clinical trials are needed to confirm this hypothesis. The cardiovascular and metabolic protective effects of plants of the Apiaceae family, with their potential role in the prevention and/or reduction of AS development, are summarised in Table 4.

#### 2.4.4. The Plants of the Lamiaceae Family

*Melissa officinalis* L., known as Lemon balm (LB), is a plant of the Lamiaceae family used in European traditional medicine in combination with other plants such as *Tilia europaea* L., *Crataegus oxyacantha* L., and *Achillea millefolium* L. to treat hypertension. To our knowledge, no information on treating AS has been reported concerning these three plants. The leaves of *Melissa officinalis* L. are used to improve cardiac function in the Lebanese population and have antioxidant properties [132,133].

Michalikova et al. [134] worked with its major polyphenol component, Rosmarinic Acid (RA), harvested from LB, which was administered in water suspension to HFFD-induced metabolic syndrome rats (100 mg/kg/day for 3 weeks). RA significantly decreased BP (*** *p* < 0.001), glycaemia (*** *p* < 0.001), IL-1β serum concentrations (* *p* < 0.05), and lipid peroxidation markers, such as Thiobarbituric Acid Reactive Substance (TBARs) (* *p* < 0.05). RA supplementation increased endothelium-dependent vascular relaxation (* *p* < 0.05), which was blunted by HFFD. Glucose tolerance test results demonstrated an antihyperglycemic effect in the same model (*** *p* < 0.001), which may correspond to improved insulin sensitisation or effective control of intestinal glucose absorption. Polyphenolic compounds are known to regulate metabolic disorders and inflammation associated with cardiovascular diseases. They are implicated in Lemon balm extract AS activity, and their mechanisms of action might involve the inhibition of liver phosphoenolpyruvate carboxykinase and increased glucose intake in muscle via GLUT4 [134].

In in vitro models of extracellular elastin and collagen fibre sheets, Yui et al. [135] studied the anti-glycation activity of 681 hot aqueous plant extracts before conducting a clinical study in healthy subjects. Among the extracts, the LB leaf extract was selected for its ability to inhibit pentosidine formation (an AGEs biomarker, IC_50_ = 0.24 mg/mL), suggesting a potential anti-AGE activity on serum albumin-fructose. Indeed, the LB extract and the isolated RA inhibited the increased elastin fibre sheet fluorescence (AGEs at 2.2 mg/mL, * *p* < 0.05). Moreover, collagen fibre treatment with RA suppressed their colouration in a concentration-dependent manner (27.8 to 222 µM, 0.01 < *p* < 0.05). Thus, an open-label, parallel-group comparative trial was conducted in 28 healthy Japanese women and men drinking LB leaf beverage (200 mL/day for 6 weeks). LB consumption in human subjects decreased PWV in the treated group compared to the control group (** *p* = 0.007) [135]. Nevertheless, an adverse effect, including creatinine reduction (* *p* < 0.05), was observed with the treatment. This finding suggested that LB reduced AS parameters in healthy humans, but further clinical research enrolling human hypertensive and/or type 2 diabetic patients is needed to confirm these benefits. The treatment with LB leaf aqueous extract reduced AGEs, one of the main factors in AS progression. The decrease in pentosidine serum levels in vivo could also attenuate oxidant and inflammatory processes, which are implicated in the induction of vascular rigidity, by limiting cell migration and VSMC proliferation. As AGE-associated oxidative stress may interact with Ang II signalling, the antioxidant effects of LB extract could explain the reduction of BP and the enhancement of endothelial function. Therefore, LB extract and RA may act on RAAS and pancreatic β-cells to control glucose homeostasis and reduce oxidative stress and inflammation. These effects could be linked to anti-AS activity and vascular wall protection from the deleterious hyperglycaemia-associated ROS and proinflammatory molecules.

*Salvia miltiorrhiza* Bunge, known as Red sage or Danshen, is another interesting plant of the Lamiaceae family, used in combination with KRG for AS management in human subjects. Indeed, the dried rhizome is used in Chinese traditional medicine to treat cardiac and vascular disorders such as coronary heart disease, myocardial infarction, and atherosclerosis [136].

Preclinical studies suggested the cardiovascular benefits of its use. Wu et al. [137] assessed the protective effect of a combination of four metabolites, SABP [Danshensu (DSS: 5 mg/kg/day) + Salvianolic acid A (Sal-A: 0.233 mg/kg/day) and C (Sal-C: 10 mg/kg/day) + Protocatechuic aldehyde (PAL: 17 mg/kg)], isolated from aqueous Red sage extract on SHR for 8 weeks. SABP decreased SBP/DBP (0.001 < *p* < 0.05) and recovered thoracic aorta adventitial fibroblasts with inhibition of VSMC hypertrophy and proliferation, and decreased NF-κB activity and IL-6 production (*** *p* < 0.001). In addition, SABP reduced pro-fibrotic indicators such as TGF-β1, Collagen I, and CTGF (*** *p* < 0.001) in aortas from SHR. These results indicated the reduction of factors contributing to AS development. Concerning the deeper mechanisms of action, an in vitro study conducted by Xu et al. [138] in endothelial cells subjected to shear stress showed that Danshen extract restored cell viability (* *p* = 0.023) and improved metabolically dependent cell viability (** *p* = 0.004) after H_2_O_2_ damage. These data corroborate the antioxidant effect of SABP, which is characterised by reduced vascular ROS and NOX1, NOX2, and NOX4 activity/expression (*** *p* < 0.001). Huang et al. [139] studied isolated bioactive compounds from Danshen (50, 100, and 200 mg/kg for 6 weeks) in STZ-induced diabetic rats. Salvianolic acid B reduced blood glucose, cholesterol, muscle and hepatic glycogen (* *p* < 0.05), and increased the insulin sensitivity index (* *p* < 0.05) in rats.

In a clinical study by Lin et al. [140], an herbal preparation (root water extract of Ginseng and Danshen, 250 mg each) was evaluated in 24 young healthy humans in a 72-h double-blind, placebo-controlled, randomised study. The treatment reduced cf-PWV (* *p* < 0.05) in the treated group, associated with IL-6 reduction (* *p* < 0.05), suggesting anti-inflammatory properties of Danshen with KRG

Based on these data, Danshen’s anti-AS activity involves decreasing inflammation. Diabetes and hypertension-associated inflammation lead to vascular wall remodelling via proinflammatory cell adhesion, which might modify collagen and elastin properties. Danshen extract treatment might restore arterial compliance and could increase glycogen synthesis, reducing circulating glucose. These effects help to reduce arterial inflammation and oxidative stress in diabetes and hypertensive patients.

The cardiovascular and metabolic protective effects of plants of the Lamiaceae family, with their potential role in the prevention and/or reduction of AS development, are summarised in Table 5.

#### 2.4.5. The Plant of the Amaryllidaceae Family

*Allium sativum* L., also known as Garlic, belongs to the Amaryllidaceae family and is recognised worldwide for treating cardiovascular disorders associated with hypertension. In African traditional medicine, bulb decoction is effective against diabetes in Morocco, whereas honey mixed with macerated crushed bulb was used to treat hypertension in Togo [141].

Preclinical studies conducted by Hasimun et al. [142] in HFFD-induced hypertensive rats have demonstrated that oral intake of fermented Garlic (50, 100, and 200 mg/kg/day for 28 days), called “black Garlic”, decreased, from the 14th day of treatment, both BP (* *p* < 0.05) and PWV (* *p* < 0.05) in a dose-dependent manner, indicating AS reduction in the treated animals. The treatment increased serum NO levels (* *p* < 0.05), suggesting NO involvement in the cardiovascular effects of Garlic in this hypertension model. Saka et al. [143] studied the aortic effects of Garlic aqueous extract (50, 100, and 150 mg/kg/day for 3 weeks) in HSD-induced hypertensive Wistar rats with vascular remodelling. Oral treatment reduced tunica media/adventitia/intima wall thickness (* *p* < 0.05) and improved aortic blood flow (* *p* < 0.05).

Concerning clinical trials, a randomised double-blind clinical study conducted by Turner et al. [144] on 75 healthy normolipidemic men and women treated with Garlic powder tablets (230 mg, twice a day for 12 weeks) demonstrated that the treatment did not change PWV (*p* = 0.88), with side effects for twelve patients (eructation after 15 min of consumption), Garlic odour for five patients, and flatulence for three patients. The work of Gruenwald et al. [145], using aqueous ethanol extract of aged Garlic (1 mL twice a day for 84 ± 3 days) in 57 healthy middle-aged men and women in a placebo-controlled clinical trial, concluded that the treatment reduced AIx (* *p* = 0.028) and DBP (* *p* = 0.038), without significant modification of the SBP (*p* = 0.056). AS reduction was associated with a decrease in blood TG levels (* *p* = 0.022) in treated subjects. Nevertheless, adverse effects such as back pain, common cold, Alanine transaminase (ALT) increase, knee joint mobilisation, and vomiting were observed in 9% of the patients. Breithaupt-Grögler et al. [146] measured the effect of Garlic on vascular elasticity by enrolling 101 healthy elderly humans treated with Garlic powder preparation (≥300 mg/day) for more than two years before the study. This long-term daily consumption lowered PWV (*** *p* < 0.0001) and increased aortic elasticity, measured by the Elastic Vascular Resistance (EVR) value, according to age. Indeed, the most significant EVR increases (0.0001< *p* < 0.0016) were detected in the oldest treated participants enrolled. These findings substantiate the benefit of consuming Garlic regularly to prevent and/or treat AS.

By referring to these results, Garlic reduced AS by decreasing BP and TG, which are AS predictive factors. Concomitant antihyperglycemic and antihypertensive activities are associated with enhanced NO bioavailability and the prevention of arterial vascular wall remodelling. Garlic increased aortic elastin production, which could enhance vascular compliance and might prevent AS-related diabetes and/or hypertension. The biological mechanisms may include the inhibition of VSMC proliferation and migration through interaction with Ang II-associated PDGF and NF-κB pathways, which are involved in vascular inflammation, NO signalling activation, and triggering elastin synthesis. The cardiovascular and metabolic protective effects of *Allium sativum* L., with its potential role in the prevention and/or reduction of AS development, are summarised in Table 6.

#### 2.4.6. The Plant of the Theaceae Family

*Camellia sinensis* L. (Kuntze), also known as Green or Black Tea, belongs to the Theaceae family. Its leaves are used in Chinese and Indian traditional medicine to increase diuresis, control glycaemia, and enhance heart function [147].

A preclinical study reported by Szulińska et al. [148] was conducted by mixing tea extract (2 and 4 g/kg) with diet and administered orally for 42 days in the NaCl-induced hypertensive Wistar rat model. Tea supplementation lowered SBP (** *p* = 0.0066) and DBP (** *p* = 0.0033), with reductions in plasma TNF-α concentration (** *p* = 0.0095) and increases in plasma total antioxidant status (TAS) (*** *p* = 0.0001). The reduction of inflammation and oxidative stress by Green Tea in this model could suggest a biological interaction with factors associated with AS.

As a daily beverage consumed by most of the Chinese population, Li C-H et al. [149] analysed the association of drinking tea with the state of vascular function in humans. The subjects that drank the highest quantities of tea (>450 mL of tea per day for at least one year) exhibited the most significant reductions in AS indicators, such as ba-PWV (*** *p* < 0.001), suggesting a dose-dependent effect in AS prevention. In a randomised double-blind placebo-controlled trial (NCT02627898), Quezada-Fernández’s [150] study with 20 normotensive type 2 diabetic patients, who took 400 mg of decaffeinated Green Tea extract daily for 12 weeks, reported that this treatment reduced AIx (* *p* = 0.04) in the treated group without affecting other metabolic parameters or sRAGE serum levels. Green Tea is rich in flavonoids such as catechins; their mechanisms of action include interaction with catecholamine signalling, increased vasodilation via improved endothelial function, vascular wall protection via inhibition of sodium-induced extracellular matrix modifications leading to AS and wall thickening [150].

As mentioned in these studies, although Green Tea consumption had no effect on the BP and metabolic profile of diabetic patients, AS was reduced. Antioxidant mechanisms could act via pathways other than AGEs. Thus, Green Tea supplementation improved arterial function, which in turn would legitimise its use in preventing oxidative stress associated with diabetes- and hypertension-induced vascular stiffness. Regarding the clinical study results, Green Tea consumption alone might be more affective in AS prevention.

The cardiovascular and metabolic protective effects of *Camellia sinensis* L. (Kuntze), with its potential role in the prevention and/or reduction of AS development, are summarised in Table 7.

#### 2.4.7. The Plant of the Caricaceae Family

*Carica papaya* L., commonly called Papaya, is a fruit tree belonging to the Caricaceae family. The leaves have been used in traditional medicine to treat dengue fever, malaria, and cancer, and to facilitate digestion [151].

In the context of AS, Hasimun et al. [152] treated Wistar rats fed a HFFD. The Papaya extract (5, 10, and 20%), mixed with nori and administered orally to treat animals for 21 days, restored BP and PWV (* *p* < 0.05) to normal values compared to non-treated animals, indicating AS reduction in this model. Concerning the antihyperglycemic effect, Juárez-Rojop et al. [153] assessed the leaf aqueous extract (0.75, 1.5, and 3.0 g/100 mL), administered orally to streptozotocin-induced diabetic rats for 30 days. Treatment decreased blood glucose, cholesterol, and Triacylglycerol (TAG) levels (*** *p* < 0.001), and increased plasma NO levels, which could impact vascular function in treated animals. Brasil et al. [154] studied the antihypertensive effect of methanolic extract of Papaya (100 mg/kg twice a day, for 30 days) in Wistar rats and SHR. In this context, the extract decreased Mean Arterial Pressure (MAP) by inhibiting the ACE enzyme and restoring the arterial baroreflex mechanism, which regulated BP (** *p* < 0.01) in treated animals. As mentioned, isolated flavonoids from Papaya leaf, such as nicotiflorin, rutin, clitorin, and manghaslin, possess anti-ACE effects that might confer the observed biological activities [154]. Directly assessing the arterial function should corroborate the positive results obtained using *Carica papaya*.

This study demonstrated the antihypertensive action of this plant by stabilising BP in HFFD-induced hypertension in rats, and its protective effects against metabolic disorders, which are deleterious to the vascular system. Even if aortic mechanisms are not well understood, by targeting glycemia and dyslipidaemia, Papaya leaf extract might reduce inflammation- and oxidative stress-induced AS, and increasing plasma NO levels could target numerous cellular and molecular triggers in the vascular and renal systems.

The cardiovascular and metabolic protective effects of *Carica papaya* L., with its potential role in the prevention and/or reduction of AS development, are summarised in Table 8.

#### 2.4.8. The Plant of the Cucurbutaceae Family

*Citrullus lanatus* (Thunb.) Matsum. & Nakai, or Watermelon, is a member of the Cucurbitaceae family and this plant has beneficial effects on vascular function.

A pilot study was conducted by Figueroa et al. [155], testing L-Citrulline/L-Arginine (1.35 g/0.65 g twice a day) for 2 × 6 weeks, separated by a 4-week washout period, in nine prehypertensive patients. Treatment decreased the amplitude of the aortic reflected wave by decreasing brachial pulse pressure (** *p* = 0.01), aortic SBP (** *p* = 0.01), and AIx (* *p* = 0.03). Another study in 14 middle-aged prehypertensive or grade 1 hypertensive patients, taking daily 6 g of L-Citrulline/L-Arginine in a 2:1 ratio for 6 weeks, with a 2-week washout period, reported a decrease in BP (* *p* < 0.05) and AIx (** *p* < 0.01) [156]. A 6-week randomised double-blind crossover study, using 6 g of L-Citrulline + 2 g of L-Arginine administered orally in 13 middle-aged obese hypertensive patients, demonstrated that AS parameters, such as AIx and pulse pressure, which both increased during the Cold Pressor Test, were decreased by Watermelon supplementation (* *p* < 0.05) [157]. Considering these data, enhancing arterial function may contribute to changing the dynamic components of AS.

Thus, another form of ageing-induced AS occurs in postmenopausal women. In a randomised cross-over study, 12 women taking L-Citrulline/L-Arginine 4:2 (6 g per day, divided into three doses, for 6 weeks), exhibited a decrease in ba-PWV (** *p* < 0.01) and the second systolic peak rSBP2 (** *p* < 0.01), compared to the placebo group, reflecting AS reduction in these patients [158]. Figueroa et al. [159] assessed a Watermelon supplementation in 16 obese hypertensive men during a 14-day randomised double-blind placebo-controlled study. The ba-PWV (** *p* < 0.01) decreased with the treatment. According to Fujie et al. [160], in a randomised crossover pilot study focused on 12 healthy young females treated with 90 g of juice extract and followed for 90 min, a significant decrease in Femoral–Ankle PWV (fa-PWV) was observed after 30 min of treatment intake until the end of the protocol (* *p* < 0.05), with an increase in plasma NO concentration starting after 60 min (* *p* < 0.05). Watermelon prevented high BP and enhanced vascular function via NO pathways. Its effectiveness has been proven in both hypertension and metabolic syndrome. As L-Arginine and L-Citrulline are NO precursors, increasing their cellular concentration could promote NO-mediated vascular activity. Since Watermelon fruit extract can reduce both BP and PWV and also increase NO-induced vasodilation, it could alleviate inflammation and oxidative stress associated with AS in hypertension.

The cardiovascular and metabolic protective effects of *Citrullus lanatus* (Thunb.) Matsum. & Nakai, with its potential role in the prevention and/or reduction of AS development, are summarised in Table 9.

#### 2.4.9. The Plant of the Zingiberaceae Family

*Curcuma longa* L., known as Turmeric or Indian Saffron, is a member of the Zingiberaceae family and has been widely used both as a spice and as a remedy in Asian traditional medicine. An ethnopharmacological survey conducted by Bussmann et al. [161] reported the use of this plant to treat diabetes and to stimulate the blood circulatory system of the Colombian population.

Apart from its cardiovascular effects in combination with other plants, Turmeric has a potential action against AS. For this purpose, Hasimun et al. [162] studied the vascular effect of Turmeric rhizome ethanolic extract (50, 100, and 200 mg/kg) in rats fed a HFFD for 28 days. The treatment decreased BP and PWV (* *p* < 0.05) in a dose-dependent manner. Moreover, NO involvement was shown to be related to the arterial activity of the plant extract. The biological activity of curcumin (a polyphenol isolated from *Curcuma longa*) was assessed by Fleenor et al. [163] in C57BL/6N elderly mice for 4 weeks, resulting in PWV (*p* < 0.05) restoration to values comparable to those of young animals. ACh-induced carotid artery relaxation was improved by the treatment (* *p* < 0.05), with an enhancement in aortic Collagen I (*p* < 0.05) and a decrease in AGE expression (* *p* < 0.05) in treated mice. NO-dependent dilation and Manganese SOD (Mn-SOD) enzyme expression were also increased (* *p* < 0.05), whereas the level of p67^phox^, a subunit of pro-oxidant NOX2, was reduced (* *p* < 0.05). A polyphenol-effective antioxidant activity reverses the occurrence of ageing-induced AS and arterial dysfunction. A review presented by Rege et al. [164] suggested that the presence of the methylene group (-CH_2_-) in its keto form conferred to curcumin its antioxidant effect by sharing a hydrogen atom to scavenge free radicals. Another study, conducted by Nakmareong et al. [165], using 50 or 100 mg/kg of Tetrahydrocurcumin (THC) in L-NAME-induced hypertensive rats for 5 weeks, demonstrated that THC increased the midwall radius of arteries (* *p* < 0.05), with subsequent vascular compliance amelioration (*** *p* < 0.001) and enhanced functional elastic radius values of arteries. These effects were associated with an oxidative stress reduction. THC is a metabolite sharing the same chemical structure as curcumin, but is more powerful than its parent molecule, due to the presence of hydrogenation at the double bond positioned on the central seven-carbon chain. Sangartit et al. [166] evaluated the vascular effect of THC (50 and 100 mg/kg for 8 weeks) in cadmium-induced aortic damage, which is associated with hypertension and oxidative stress in mice. The THC treatment reduced AS by lowering both aortic MMP-2 and MMP-9 levels (*** *p* < 0.001) and increasing eNOS-associated vasodilation (*** *p* < 0.001) and elastin formation (* *p* < 0.05) in hypertensive animals. According to Kuroda et al. [167], on antidiabetic research in diabetic KK-Ay/T mice, a 4-week diet containing Turmeric rhizome ethanolic extract (0.2 or 1.0 g/100 g dose) resulted in reduced blood glucose (*p* < 0.05) in treated groups. In an in vitro study, using human preadipocytes treated with 5 µg/mL or 10 µg/mL of the ethanolic extract, strong ligand-binding to PPAR-γ receptors (*p* < 0.05) was exhibited [167].

In a randomised, double-blind, controlled clinical trial conducted by Srinivasan et al. [168] on 114 type 2 diabetes mellitus patients with a 3-month Turmeric supplementation (400 mg), they observed decreased AS parameters, including cf-PWV (*** *p* < 0.001), ba-PWV (**** *p* < 0.0001), and aortic AIx (** *p* = 0.007), suggesting Turmeric had arterial de-stiffening activity. The treatment was well tolerated, except for a reduction in plasma alkaline phosphatase (* *p* = 0.033). Only two treated patients presented adverse effects, such as an increase in stool frequency and upper abdominal pain. Indian saffron ethanolic extract and curcumin de-stiffening activity decreased AGEs and aortic MMP production, and increased NO production. In line with these results, by reducing oxidative stress associated with a diabetic state, bioactive compounds such as curcumin or Curcuma longa extract prevented aortic vascular thickness and dysfunction. Apart from reducing diabetes vascular risk factors, Turmeric extracts restored BP and elastin activity both in diabetic and hypertensive animal models. Thus, using Indian saffron as a food supplement could prevent AS and increase vascular relaxation due to the restoration of aortic distensibility. The cardiovascular and metabolic protective effects of *Curcuma longa* L., with a potential role in the prevention and/or reduction of AS development, are summarised in Table 10.

#### 2.4.10. The Plant of the Clusiaceae Family

*Garcinia mangostana* L., called Mangosteen, is part of the Clusiaceae family. In Thai indigenous medicine, pericarp has been used against skin infections, diarrhoea, and wounds [169]. A study carried out by Wihastuti et al. [170] in hypercholesterolemic rats treated with 200, 400, or 800 mg/kg of pericarp ethanolic extract for 2 months demonstrated the following: decrease of aortic VCAM-1 expression in a dose-dependent manner (* *p* = 0.027, * *p* = 0.018 and ** *p* = 0.009), thinning of perivascular adipocyte tissue (* *p* = 0.046, with the highest dosage), as well as reduction of intima-media thickness (*** *p* < 0.001). Thus, inflammation associated with aortic wall thickness progression is decreased with the treatment. In the case of oxidative stress and inflammation, a study conducted by Boonprom et al. [171] on L-NAME hypertensive rats demonstrated a reduction in SBP, wall thickness, wall thickness/lumen ratio, aortic superoxide anion production, aortic p47^phox^ NOX2 subunit, and iNOS enzyme expression, as well as circulating TNF-α (* *p* < 0.05) after 5 weeks of oral treatment with 200 mg/kg/day of pericarp aqueous extract. The xanthones and flavonoids, such as α-mangostin and catechins isolated from Mangosteen pericarp extract, contribute to reducing vascular inflammation and oxidative stress by inhibiting arachidonic acid signalling in neutrophil cells and H_2_O_2_-induced senescence in endothelial cells [172,173]. As flavonoids are acknowledged to inhibit the ACE enzyme and are a part of these anti-inflammatory and antioxidant properties, inhibition of RAAS could involve lowering AS through this plant extract treatment.

Concerning clinical studies, as demonstrated by Maejima et al. [174] in a twelve-week placebo-controlled, double-blinded, randomised trial performed on 40 healthy Japanese women, 12 weeks of treatment with 200 mg/day of Mangosteen pericarp water extract reduced Arterial Pressure Index (API) (* *p* < 0.05) and Arterial Velocity Pulse Index (AVI) (** *p* < 0.010) in the treated group without inducing adverse effects. The extract amplified aortic vasodilation, and this effect was associated with a decrease in plasma pentosidine (* *p* < 0.05), which is related to AGE formation. Improving vascular function was linked with a decrease in glycated haemoglobin (Hb1Ac), total cholesterol, and LDL-C (* *p* < 0.05). By considering these results, Mangosteen AS reduction mechanisms involve mainly antioxidant and anti-inflammatory effects. The decreased values of metabolic parameters might reflect aortic remodelling and the regression of inflammation-associated vascular dysfunction, and prevent hypertension. Mangosteen could inhibit the adhesion of immune proinflammatory cells to vascular walls, limiting cell proliferation and migration. Considering its effects on the NOX subunit and iNOS activity and expression, the plant extract’s main advantages concern arterial wall structure restoration, which could reduce diabetes-induced AS. The cardiovascular and metabolic protective effects of *Garcinia mangostana* L., with its potential role in the prevention and/or reduction of AS development, are summarised in Table 11.

#### 2.4.11. The Plant of the Cactaceae Family

*Hylocereus undatus* (Haw.) Britton et Rose, also called Dragon fruit or Red pitaya, of the Cactaceae family, has been known to have antioxidant properties.

The vascular effect of fruit pulp aqueous extract was assessed by Swarup et al. [175] in STZ-induced diabetes in rats receiving 250 or 500 mg/kg/day of extract by gavage for 5 weeks. Apart from lowering blood glucose levels (* *p* < 0.05), the extract reduced SBP and PWV (* *p* < 0.05). Fruit pulp extract activity was linked to a decrease in MDA enzyme activity and improvement of antioxidant defence, characterised by increased SOD and Total Antioxidant Capacity (TAC) (* *p* < 0.05) at the 500 mg/kg dose. These results demonstrated the role of Red pitaya antioxidant properties in reducing AS.

Concerning the bioactive compounds involved in the beneficial effects of this plant, Cheok et al. [176] evaluated betalain, a tyrosine-derived pigment, in 19 healthy human volunteers consuming 24 g of whole Dragon fruit (containing 33.41 mg of betalain), during an acute, randomised, controlled, crossover, double-blind clinical trial, followed by 14-days of consumption. After 3 h of intake, PWV decreased significantly (** *p* = 0.003), but differences from the control group were not observed after 2 weeks (*p* = 0.086). For AIx, no significant trend was found acutely (*p* = 0.357), but the reduction was statistically significant (* *p* = 0.020) at the end of the protocol. An increase in FMD, compared with the placebo group, was observed from 2 h after intake, and the effect was maintained until 14 days after intake (* *p* = 0.039). Dragon fruit extract induces acute enhancement of arterial function and AS reduction in healthy conditions [176].

Red pitaya fruit pulp water extract reduces oxidative stress and AS in a rat model of metabolic syndrome. Consumption in healthy conditions improves AS-dependent parameters. Thus, the use of Dragon fruit extract could contribute to alleviate or prevent oxidative stress-induced AS in metabolic disorders or other stress conditions involved in cardiovascular diseases. The cardiovascular and metabolic protective effects of *Hylocereus undatus* (Haw.) Britton et Rose, with its potential role in the prevention and/or reduction of AS development, are summarised in Table 12.

#### 2.4.12. The Plant of the Myrsinaceae Family

*Labisia pumila* (Blume) Fern. -Vill. *Var Alata*, called Kapic Fatimah, is a plant of the Myrsinaceae family traditionally used in Asia to facilitate delivery and to improve women’s sexual desire.

According to Al-Wahaibi et al. [177], in a study using ovariectomized rats treated with 17.5 mg/kg/day for 3 months, the plant aqueous extract reduced wall thickness increased by ovariectomy (* *p* < 0.05) and improved aortic elastin structure (* *p* < 0.05), which was reduced in ovariectomized (OVX) non-treated female rats compared to normal (non-ovariectomized) rats. This model exhibited AS induced by menopause and demonstrated the beneficial effects of Kapic Fatimah in enhancing elastin structure and wall thickness, which are structural components of vascular stiffness. For antidiabetic activity, Mansor et al. [178] evaluated the effect of 50 mg/kg of Kapic Fatimah aqueous extract in rats with polycystic ovary syndrome, which is characterised by insulin resistance. The 4–5 weeks of treatment increased glucose uptake and insulin sensitivity. The effect was associated with an increase in Peroxisome Proliferator-Activated Receptor gamma (PPAR γ) protein levels (* *p* < 0.05). As insulin resistance is involved in AS development via increased collagen synthesis [3], triggering insulin pathways could be an advantage of using Kapic Fatimah in AS management. The reviews by Abdullah et al. [179] and Silva [180] concluded that phytoestrogens, such as isoflavones, derived from this plant and well known for their vascular protective effects, decreased BP and enhanced vascular function. Enhancement of vasorelaxation resulted in endothelium and VSMC stimulation, which, in turn, induced cell hyperpolarisation (interaction with potassium channels) or production of Endothelium-Derived Hyperpolarising Factors (EDHFs) via NO/cyclic Guanosine Monophosphate (cGMP) signalling [180].

The restoration of elastin structure and vascular wall integrity permits arterial compliance and distensibility. As menopause is associated with vascular dysfunction and increased risk of cardiometabolic diseases, the biological properties of the plant extract evidenced in these studies might contribute to controlling vascular inflammation and oxidative stress-induced AS by modulating cellular glucose uptake and enhancing not only insulin sensitivity but also NO production via downstream proteins and gene regulation. Moreover, treatment and prevention of AS could be effective in cases of diabetes and hypertension. The cardiovascular and metabolic protective effects of *Labisia pumila* (Blume) Fern. -Vill. *Var Alata*, with its potential role in the prevention and/or reduction of AS development, are summarised in Table 13.

#### 2.4.13. The Plant of the Thymelaeaceae Family

*Phaleria macrocarpa* (Scheff.) Boerl., called Mahkota dewa or God’s crown, is a plant that belongs to the Thymelaeaceae family. The leaves and fruit are most commonly used in traditional medicine to reduce glycaemia, BP, and blood cholesterol. After being cut into pieces and dried, the fruit plant preparation included either decoction with other plant leaves or infusion with hot water. The remedy is taken three times a day in Malaysian traditional medicine [181]. The cardiovascular activity of fruit aqueous extract (250, 500, and 1000 mg/kg) was studied by Altaf et al. [182] in SHR, with the extract administered orally for 3 weeks. The treatment decreased MAP, heart rate, and PWV (** *p* < 0.01) in the group receiving 1000 mg/kg. The treatment lowered AS, and the plant extract reduced calcium-induced aortic contraction (* *p* < 0.05), indicating its involvement in regulating cellular calcium homeostasis by inhibiting external calcium entry into the cell and/or by releasing calcium from the endoplasmic reticulum into the cytoplasm. The polar (chromatography) fraction, rich in flavonoids (kaempferol 3-O-β-glucuronide, mangiferin, gallic acid, and rutin), was considered to be involved in the cardiovascular beneficial effects of God’s crown in rats. Indeed, these bioactive compounds regulated intracellular calcium influx in the aorta. Because the fraction rich in kaempferol induced greater vasodilation, this confirmed that this molecule was responsible for vascular endothelium activity and AS reduction [182]. Daud et al. [183] evaluated the effects of the methanolic extract in SHR. The treatment with this fruit decreased blood glucose in treated animals (* *p* < 0.05).

Finally, a clinical trial conducted by Rizal et al. [184] on 40 elderly hypertensive patients demonstrated the ability of treatment with God’s crown fruit to reduce BP. Based on these data, use of the plant fruit aqueous extract alleviated both AS progression and vascular disease-induced or high BP-associated hypertension. By interfering with the NO-dependent vasodilation and blood sugar homeostasis, the vascular protection properties of the aqueous extract might therefore prevent or treat arterial inflammation, oxidative stress, and vascular dysfunction/wall thickening that lead to AS in diabetic and hypertensive patients. The cardiovascular and metabolic protective effects of *Phaleria macrocarpa* (Scheff.) Boerl., with its potential role in the prevention and/or reduction of AS development, are summarised in Table 14.

#### 2.4.14. The Plant of the Phyllanthaceae Family

*Phyllantus emblica* L., or *Emblica officinalis* Gaertn, called Amla or Indian Gooseberry, belongs to the Phyllanthaceae family. In Ayurvedic medical practice, the people of India use Amla fruit to delay the organ ageing process and to protect the nervous system. It is useful in controlling blood sugar and increasing urine production [185].

Patil et al. [186] evaluated Amla fruit ethanolic extract in a high-fat diet rat model administered the extract orally (100 mg/kg/day) for 21 days. Treatment increased aortic NO and decreased MDA enzyme levels (**** *p* < 0.0001). Indian Gooseberry extract diminished tunica intima layer thickness (** *p* = 0.002) and restored elastin structure. It enhanced arterial elasticity, and this effect is associated with an increase in NO bioavailability and a reduction in oxidative stress. Srinivasan et al. [187] evaluated flavonoids isolated from Amla fruit extract in STZ-induced diabetic rats. Quercetin treatment decreased blood glucose, urine sugar levels, cholesterol, and raised insulin and haemoglobin plasma concentrations. The quercetin mechanism might decrease α-glucosidase enzyme activity and/or increase insulin sensitisation by binding to the PPARγ receptor.

A double-blind, placebo-controlled, crossover clinical study conducted by Pingali et al. [188], using 250 mg of fruit water extract (twice daily) for 14 days in 12 healthy humans subjected to a Cold Pressor Test to induce arterial stiffness, showed that Amla extract decreased AS by reducing BP and AIx (* *p* < 0.05) in treated subjects compared to the placebo group. Abdominal discomfort and headache were reported by two volunteers as adverse effects during the study. Another randomised, double-blind, placebo-controlled study was conducted by Usharani et al. [189] using standardised aqueous extract rich in tannins (Emblicanin-A, Emblicanin-B, Pedunculagin, and Punigluconin) in 59 humans with metabolic syndrome. Dosage was taken orally (250 or 500 mg twice a day) for 12 weeks. The treatment reduced the Reflection Index (RI) (*** *p* < 0.001) and increased plasma NO concentration (* *p* < 0.05), indicative of an improvement in endothelial vasorelaxation. Moreover, oxidative stress was decreased, and the pro-oxidant enzyme MDA and proinflammatory hsCRP were also decreased (* *p* < 0.05) by the treatment. Concerning metabolic parameters, Amla extract reduced plasma TC, LDL-C, and TG, and increased HDL-C (* *p* < 0.05). Amla improvement of arterial function is associated with the inhibition of inflammation and oxidative stress in these patients. Amla fruit aqueous extract, mainly composed of Quercetin, reduced MDA and BP and increased endothelial NO, which plays a key role in enhancing vascular relaxation and preventing AS. In addition to decreasing glycaemia and pro-oxidant proteins, Amla fruit extract could limit blood vessel inflammation progression and VSMC growth/migration during the onset of AS or in cardiovascular complications associated with diabetes and hypertension. The cardiovascular and metabolic protective effects of *Phyllantus emblica* L., with its potential role in the prevention and/or reduction of AS development, are summarised in Table 15.

#### 2.4.15. The Plant of the Moringaceae Family

*Moringa oleifera* Lam., commonly known as drumstick, belongs to the Moringaceae family. In Nigerian and Malagasy traditional medicine, the leaves, flowers, and seeds are used to alleviate symptoms of infections, to treat cough, and to reduce high blood pressure [190,191]. Aekthammarat et al. [192] studied L-NAME-induced hypertension in anaesthetised rats treated with *Moringa oleifera* leaves aqueous extract (1, 3, 10, and 30 mg/kg), administered via femoral artery injection. The Moringa treatment reduced MAP while the extract provoked ex vivo an endothelium-dependent vasodilation in rat mesenteric arteries, associated with increased NO production (* *p* < 0.05). Furthermore, we previously demonstrated that the powder of Moringa seeds (oral treatment with 750 mg/day for 20 weeks) showed a decrease in aortic oxidative stress (**** *p* < 0.0001), associated with reduced expression of NOX subunits (p22^phox^ and p47^phox^) (* *p* < 0.05), nitrosative stress, and inflammatory iNOS expression (* *p* < 0.05). Interestingly, endothelial function was also improved. This is evidenced by enhanced carbachol-induced mesenteric artery relaxation in the treated group [193]. According to Lindesay et al. [9], ageing induces arterial stiffness in rats, characterised by a reduction in aorta distension wave and by an increase in β-stiffness index. By comparison, we evaluated the vascular anti-ageing property of Moringa seeds in middle-aged Wistar rats treated with 750 mg/day for 4 weeks [194]. We evidenced that *Moringa* treatment restored aorta and mesenteric artery endothelial function (*** *p* < 0.001) in a similar manner to that observed in young rats. The mechanisms of action were associated with NO production deriving from eNOS activity improvement through signalling increase, and Arginase-1 downregulation (*** *p* < 0. 001). Indeed, Arginase-1 is involved in Ang II-induced AS while SIRT1 overexpression attenuated vascular stiffening in rodents [195,196]. Our data obtained in middle-aged rats, in agreement with these studies, demonstrated the implication of drumstick *Moringa* seed powder in increasing SIRT1 aortic expression and activity (*** *p* < 0.001) [197], suggesting potential anti-arterial stiffness activity. Concerning antihypertensive and antidiabetic activities, low doses of *Moringa oleifera* seed powder were able to reduce fasting blood sugar and glycosylated haemoglobin, increase antioxidant enzymes, and decrease proinflammatory cytokines such as IL-6 (* *p* < 0.05) [198]. There are controversial studies about the effect of *Moringa oleifera* on hypertension, depending on the animal models used. We did not find an antihypertensive effect of *Moringa oleifera* seed powder in SHR [199], but in contrast, Oyeleye et al. [200] reported a reduction in both SBP and DBP in L-NAME-induced hypertensive rats (* *p* < 0.05). By considering the mechanisms of action of *Moringa oleifera* seed powder, associated mainly with oxidative stress and inflammation reduction, drumstick seed may act by decreasing inflammatory processes and may be used in preventing and/or treating vascular remodelling associated with AS. The cardiovascular and metabolic protective effects of *Moringa oleifera* Lam, with its potential role in the prevention and/or reduction of AS development, are summarised in Table 16.

## 3. Discussion and Conclusions

Reducing AS is crucial for preventing/treating cardiovascular complications and vascular disease development associated with diabetes and hypertension. Aerobic and aquatic walking exercises, practised especially in the afternoon or the evening, have been demonstrated to reduce both BP and glycemia and prevent AS progression. Antihypertensive drugs, acting via RAAS inhibition, such as ARBs, ACEi, and DRI, are the most effective in lowering AS. Antihyperglycemic and metabolism-effective drugs are also able to reduce AS through numerous mechanisms: by decreasing oxidative stress, by reducing inflammatory processes, by modulating Collagen Type I and TGF-β protein expression, as well as by improving endothelial function. Given the multifactorial origins of vascular stiffness, rats or mice fed a HFFD or STZ-induced diabetes are the animal models most commonly used to study AS. Most preclinical studies cited in this review used a model of rats that become hypertensive by HFFD. It is a model of vascular stiffness preceded by hypertension associated with inflammation, oxidative stress, and decreased NO bioavailability. This is a pertinent model because it is characterised by an increased PWV associated with vascular stiffness and fibrosis [201,202]. A large portion of the studies cited in this review used this model to evaluate the vascular effect of plant, plant extract, or natural compound treatments; however, the authors mainly focused their analyses on the NO pathway instead of exploring vascular remodelling aspects, except for a study on isolated molecules from *Melissa officinalis* L. extract, whose biological effects targeted hypertension, blood sugar, and pro-oxidant agents involved in damage to arterial function and structure, with proven anti-rigidity properties [135]. Moreover, the use of STZ in rodents is also pertinent because this treatment accelerates vascular stiffness by inducing endothelial dysfunction and vascular wall structural changes, marked by an altered vascular collagen/elastin ratio, leading to premature vascular ageing. This animal model is also employed for the research on vascular complications in diabetes [203,204]. Plants such as *Panax ginseng* C.A. Meyer, *Panax quinquefolius* L., and *Hylocereus undatus* (Haw.) Britton and Rose were tested in STZ-treated animals, but more data were obtained on the mode of action of KRG treatment with plants, plant extracts, or plant fractions containing ginsenosides as bioactive molecules.

**Figure 2:**
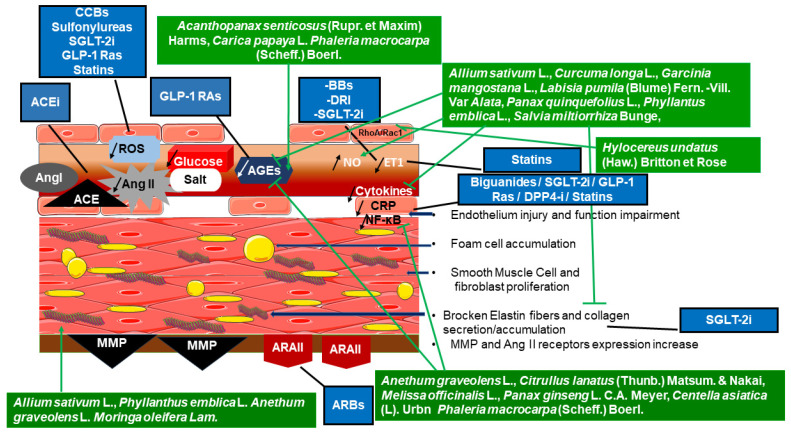
Drugs’ and plants’ general molecular and cellular targets in the prevention/treatment of arterial stiffness (AS). Ang II: Angiotensin II; ACE: Angiotensin Converting Enzyme; AGEs: Advanced Glycation End-products; ROS: Reactive Oxygen Species; NO: Nitric Oxide; MMP: Matrix Metalloproteinase; ARA II: Angiotensin II receptors; CRP: C-reactive protein; NF-κB: Nuclear Factor Kappa-B; ACEI: Angiotensin Converting Enzyme Inhibitor; SGLT-2i: Sodium-Glucose Cotransporter 2 inhibitor; DPP4-i: Dipeptidyl Peptidase 4 inhibitor; GLP-1 Ras: Glucagon-Like Peptide 1 Receptor Agonists; BBS: Beta-Blockers; DRI: Direct Renin Inhibitors. ↗ = increased; ↙ = decreased; ↓ = from to.

Regarding other animal models, such as SHR, L-NAME- or HSD-treated rats, high blood pressure-dependent mechanisms develop, while a PWV increase independent of blood pressure is induced in the rodent models of ageing or ovariectomy. Research conducted with *Panax ginseng* C.A. Meyer, *Curcuma longa* L., *Anethum graveolens* L., and *Labisia pumila* (Blume) Fern. -Vill. *Var Alata* (cf. 2.4.2, 2.4.3, 2.4.9, 2.4.12) mainly focused on endothelium-dependent relaxation and on the alterations of collagen and elastin associated with oxidative stress, which would thus limit the applicability of the results to only some AS-associated factors. Among the plants described, some of the extracts and bioactive compounds have been reported to reduce both blood sugar and/or blood pressure. For some of the natural substances, the suggested antidiabetic mechanisms include increased GLUT gene expression, renal glucose excretion, and insulin sensitisation/secretion. As demonstrated in several preclinical studies conducted on animal models, as well as in the clinical trials reported by Oh et al. [110] and Rizal M. et al. [184], extracts of *Acanthopanax senticosus* (Rupr. et Maxim) Harms, *Carica papaya* L., and *Phaleria macrocarpa* (Scheff.) Boerl. could act on pancreatic β-cells to release insulin and control glucose homeostasis, similar to the effect of sulphonylureas. Considering the antihyperglycaemic and hypotensive properties of plant extracts from *Allium sativum* L., *Curcuma longa* L., *Garcinia mangostana* L., *Labisia pumila* (Blume) Fern. -Vill. Var Alata, *Moringa oleifera* Lam., *Panax quinquefolius* L., *Phyllantus emblica* L., and *Salvia miltiorrhiza* Bunge, the relaxing activity was evaluated based on the effect of the treatment on PWV and/or FMD or AIx. By improving FMD/AIx, these plant extracts may have an effect similar to that of SGLT-2 inhibitors. Indeed, interacting with the renal system to inhibit glucose reabsorption, natural molecules could contribute to alleviating hyperglycaemia’s detrimental vascular effects (Figure 2), as demonstrated by several in vivo and in vitro preclinical research and observational studies, as well as in clinical trials. In contrast, extracts of *Anethum graveolens* L., *Citrullus lanatus* (Thunb.) Matsum. & Nakai, *Melissa officinalis* L., and *Panax ginseng* C.A. Meyer, which reduce PWV, may have mechanisms of action similar to those of GLP-1 RAs and/or DPP4-i pharmacological inhibitors. Indeed, these plant extracts have potential properties to enhance glucose utilisation in the body by increasing insulin sensitivity with subsequent AS reduction. Finally, extracts collected from *Centella asiatica* (L). Urb. and *Phaleria macrocarpa* (Scheff.) Boerl. may be effective, as demonstrated in preclinical studies conducted on animal models and in two clinical trials by Jovanovski et al. [117] and Lin H.F. et al. [140]; nevertheless, more in-depth studies concerning both antidiabetic and anti-vascular stiffness would be necessary to elucidate their exact therapeutic classification in the context of AS.

Among plants reported in this review, five—including *Anethum graveolens* L., *Centella asiatica* (L). Urb., *Carica papaya* L., *Labisia pumila* (Blume) Fern. -Vill. Var. Alata, and *Moringa oleifera* Lam.—have not been clinically tested to evaluate and scientifically demonstrate their efficacy against AS in humans. On the other hand, some plants, plant extracts, or molecules isolated from *Panax ginseng* C.A. Meyer, *Melissa officinalis* L., *Citrullus lanatus* (Thunb.) Matsum. & Nakai, and *Hylocereus undatus* (Haw.) Britton and Rose, have only been tested in clinical trials with a small number of subjects included; therefore, these trials would be considered pilot studies for a qualitative assessment of effects in humans and need to be confirmed in a larger cohort and across different AS-linked pathologies to provider stronger evidence from data analyses.

In terms of study duration, most clinical trials cited in this review lasted on average 3 months, except for *Allium sativum* L. and *Camellia sinensis* L. (Kuntze), which lasted 2 years or more than 1 year, respectively. Only the treatment with *Hylocereus undatus* (Haw.) Britton and Rose had a shorter duration of 14 days. Finally, regarding gender considerations, the clinical trials that enrolled male and female healthy subjects or patients did not consider the effects of gender on their results. In contrast, the clinical trials conducted with *Acanthopanax senticosus* (Rupr. et Maxim) Harms or *Salvia miltiorrhiza* Bunge included only men, while those conducted with *Garcinia mangostana* L. focused only on women.

As each plant, plant extract, or isolated compound has its own effect on AS, using these plant extracts in combination rather than alone could potentiate their impact on the vascular wall rigidity and on increased arterial thickness (Figure 2). Besides decreasing BP and increasing NO bioavailability, Garlic (*Allium sativum* L.) and Amla (*Phyllanthus emblica* L.) reduced arterial remodelling by improving blood flow and by enhancing elastin neo-synthesis, respectively. Dill (*Anethum graveolens* L.) also increased elastin production, which might result in improved aortic distensibility (Figure 2). The prevention of AS dynamic and structural component alterations requires inhibition of vascular inflammation and oxidative stress, which are the mechanisms of action observed for Mangosteen (*Garcinia mangostana* L.) and Amla (*Phyllanthus emblica* L.) to treat AS in animals and humans.

In conclusion, despite the scarcity of studies with isolated bioactive compounds or fractions from several traditional plants, amino acids (L-Citrulline), polyphenols (Curcumin and THC), saponins (Ginsenoside Rb1), tyrosine (Betalain), and flavonoids have been demonstrated to possess anti-AS activity both in animal models and in humans. Therefore, in this review, we suggest that, through different biological mechanisms, medicinal plants could possess the potential to prevent AS, particularly by acting against several factors linked to the development of diabetes and/or hypertension, which are involved in AS induction. In other cases, natural treatments could prevent the effects of vascular ageing, which is also involved in the development of AS. The best targets to reduce vascular stiffness risks would be both lower blood sugar and a low blood pressure. In this perspective, several natural compounds from traditional plants could have concomitant antihyperglycemic and antihypertensive properties. Flora and/or bioactive compounds isolated from plants may be a potential source of remedies. Research, guided by the medicinal properties of plants, can lead to both improved and restored functions of the vascular structure by promoting more effective use of diets including these plants or plant extracts containing well characterised bioactive beneficial molecules. The positive impact could be both to better prevent and decrease the development of vascular disease and to reduce the use of conventional drugs when vascular disease is established, if natural compounds possess similar pharmacological effects with fewer adverse effects. Natural treatments from plants, when deeply analysed and studied, might be safely used as an alternative or in association with drugs for AS prevention or treatment.

## Figures and Tables

**Table 1:** Synthesis of cellular and/or animal models used in preclinical studies conducted to test the vascular, metabolic, anti-inflammatory, and antioxidant effects of eighteen plants with potential properties against arterial stiffness.

Plant Name	Preclinical Studies (Cell and/or Animal Models)
*Acanthopanax senticosus* (Rupr. and Maxim)	HFD-induced obese C57BL/6J mice
*Panax ginseng* C.A. Meyer	Aged C57BL/6J mice; VSMC; STZ-induced diabetes in rats
*Panax quinquefolius* L.	STZ-induced diabetes in mice and rats
*Anethum graveolens* L.	Aged C57BL/6J mice; Alloxan-induced diabetes in mice
*Centella asiatica* (L). Urb.	HFFD-induced hypertensive rats; SHR; L-NAME-induced hypertensive Sprague–Dawley or Wistar rats; STZ-induced diabetes in rats
*Melissa officinalis* L.	HFFD-induced metabolic syndrome in rats; In vitro collagen and elastin fibre sheets; Glycation-induced collagen colouration in vitro model
*Salvia miltiorrhiza* Bunge	SHR; Wistar Kyoto rats; in vitro endothelial cells; STZ-induced diabetes in rats
*Allium sativum* L.	HFFD-induced hypertension in Wistar rats; (NaCl 8%)-induced aortic remodelling in rats
*Camellia sinensis* L. (Kuntze)	NaCl-induced hypertensive Wistar rat model
*Carica papaya* L.	HFFD-induced hypertensive rats; STZ-induced diabetes in rats; SHR
*Citrullus lanatus* (Thunb.) Matsum. & Nakai	-
*Curcuma longa* L.	HFFD-induced hypertension in rats; Aged C57BL/6N mice; L-NAME hypertensive rats; cadmium-induced aortic damage in rats; cadmium-induced aortic damage with hypertension and oxidative stress; diabetic KK-Ay/T mice
*Garcinia mangostana* L.	Hypercholesterolemic rats; L-NAME hypertensive rats; H_2_O_2_-induced senescence in endothelial cells
*Hylocereus undatus* (Haw.) Britton et Rose	STZ-induced diabetes in Sprague–Dawley rats
*Labisia pumila* (Blume) Fern. -Vill. *Var Alata*	OVX female rats; Rats with polycystic ovary syndrome
*Phaleria macrocarpa* (Scheff.) Boerl.	SHR and Wistar Kyoto rats
*Phyllantus emblica* L.	HFD rat model; STZ-induced diabetes in rats
*Moringa oleifera* Lam.	L-NAME-induced hypertensive rats; SHR; Middle-aged Wistar rats

HFD: high-fat diet; VSMC: Vascular Smooth Muscle Cells; KK-Ay/T: Yellow Kuo Kondo; STZ: streptozotocin; HFFD: High-Fat High-Fructose; SHR: Spontaneously Hypertensive Rats; L-NAME: N-Nitro-L-Arginine Methyl Ester hydrochloride; OVX: ovariectomized.

**Table 2:** Synthesis of the pathologies or health status of the enrolled subjects in clinical trials conducted to test the metabolic, anti-inflammatory, and antioxidant effects of natural extracts from plants with potential properties against arterial stiffness.

Plant Name	Clinical Trials Yes/No	Pathology or Health Status of the Enrolled Subjects	Number of Subjects	Gender of the Subjects
*Acanthopanax senticosus* (Rupr. et Maxim) harms	Yes	Hypertensive and metabolic Disorder; Smokers	76	♂
*Panax ginseng* C.A. Meyer	Yes	Healthy humans Type 2 diabetes subjects Stable angina with coronary artery stenosis	17 25 20	♂ and ♀ ♂ and ♀ ♂
*Panax quinquefolius* L.	Yes	Hypertension and type 2 diabetes	64	♂ and ♀
*Anethum graveolens* L.	No	-	-	-
*Centella asiatica* (L.) Urb.	No	-	-	-
*Melissa officinalis* L.	Yes	Healthy subjects	28	♂ and ♀
*Salvia miltiorrhiza* Bunge	Yes	Healthy young subjects (eccentric exercise)	24	♂
*Allium sativum* L.	Yes	Healthy normolipidemic humans Healthy middle-aged or grade 1 hypertensive patients Healthy elderly humans	75 57 101	♂ and ♀ ♂ and ♀ ♂ and ♀
*Camellia sinensis* L. (Kuntze)	Yes	Healthy Chinese subjects Type 2 diabetes patients	3135 20	♂ and ♀ ♂
*Carica papaya* L.	No	-	-	-
*Citrullus lanatus* (Thunb.) Matsum. & Nakai	Yes	Prehypertension Prehypertension and hypertension Middle-aged obese patients with hypertension Postmenopausal women Obese male with hypertension Healthy young subjects	9 14 13 12 16 12	♂ and ♀ ♂ and ♀ ♂ and ♀ ♀ ♂ ♀
*Curcuma longa* L.	Yes	Type 2 diabetes patients	114	♂ and ♀
*Garcinia mangostana* L.	Yes	Healthy subjects	40	♀
*Hylocereus undatus* (Haw.) Britton et Rose	Yes	Healthy subjects	18	♂ and ♀
*Labisia pumila* (Blume) Fern. -Vill. *Var Alata*	No	-	-	-
*Phaleria macrocarpa* (Scheff.) Boerl.	Yes	Elderly hypertensive patients	40	♂ and ♀
*Phyllantus emblica* L.	Yes	Healthy human subjects subjected to cold pressor test Endothelial dysfunction, oxidative stress, systemic inflammation, and lipid profile in subjects with metabolic syndrome	15 59	♂ ♂ and ♀.
*Moringa oleifera* Lam.	No	-	-	-

**Table 3:** Cardiovascular and metabolic effects of *Acanthopanax senticosus* (Rupr. et Maxim) Harms, *Panax ginseng* C.A. Meyer, and *Panax quinquefolius* L.

Family	Name of the Plant		Effects of the Plant
Araliaceae	* 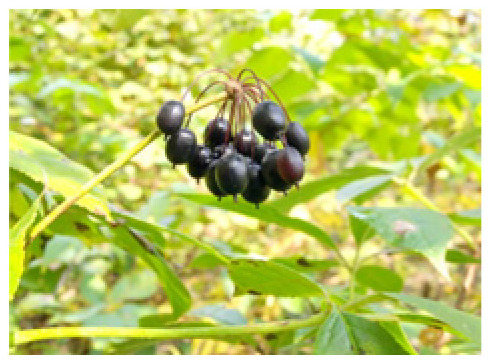 *	*Acanthopanax senticosus* (Rupr. et Maxim) Harms	Reduction of vascular stiffness (decrease in PWV and SBP values); improvement of vasodilation associated with eNOS/NO production; antihyperglycemic and antioxidant properties.
	* 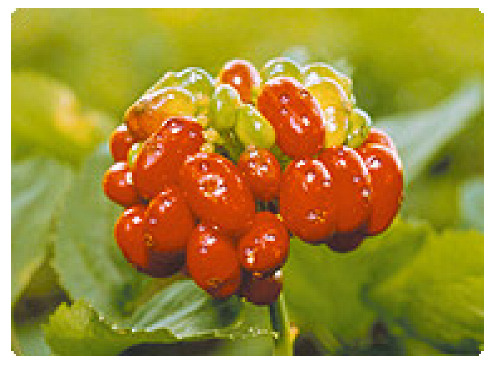 *	*Panax ginseng* C.A. Meyer	Vasodilation activity (reduction in arginase, enhanced eNOS/NO production); reduction of vascular rigidity due to ageing; antihyperglycemic and antioxidant properties.
	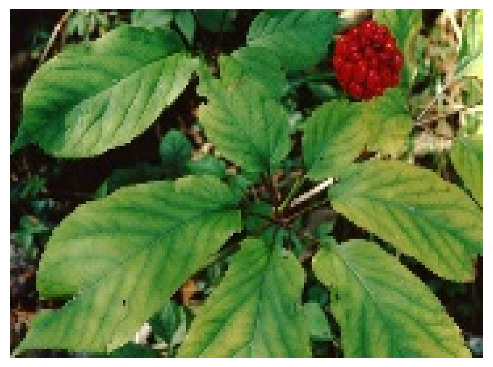	*Panax quinquefolius* L.	AS reduction; arterial relaxation enhancement and antihypertensive property; reduced blood glucose, TG, cholesterol, and insulin resistance.

PWV: Pulse Wave Velocity; SBP: systolic blood pressure; eNOS: endothelial Nitric Oxide Synthase; NO: Nitric Oxide; TG: triglycerides. The images of each plant are presented in the table and were obtained from the free picture website https://depositphotos.com, https://www.avogel.ca, https://www.shutterstock.com/ respectively.

**Table 4:** Cardiovascular and metabolic effects of *Anethum graveolens* L. and *Centella asiatica* (L). Urb.

Family	Name of the Plant		Effects of the Plant
Apiaceae	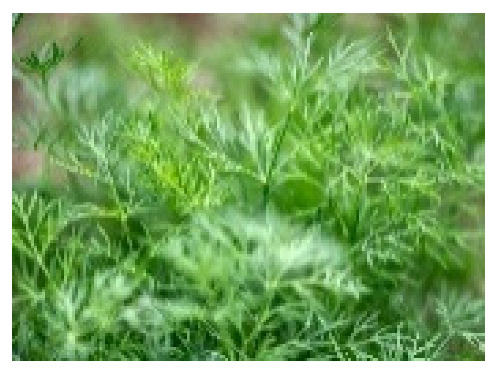	*Anethum graveolens* L.	Decrease in SBP and DBP; increase in aortic distensibility and improved endothelial function; reduced elastin degradation and improved elastic fibre neo-synthesis.
	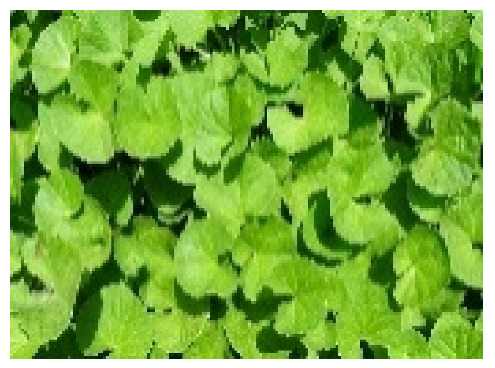	*Centella asiatica* (L). Urb	Reduction in BP and PWV; enhanced eNOS/NO production and increased vasorelaxation. Antioxidant and anti-inflammatory effects.

PWV: Pulse Wave Velocity; SBP: systolic blood pressure; DBP: diastolic blood pressure; eNOS: endothelial Nitric Oxide Synthase; NO: Nitric Oxide. The images of each plant are presented in the table and were obtained from the free picture website https://pixabay.com.

**Table 5:** Cardiovascular and metabolic effects of *Melissa officinalis* L. and *Salvia miltiorrhiza* Bunge.

Family	Name of the Plant		Effects of the Plant
Lamiaceae	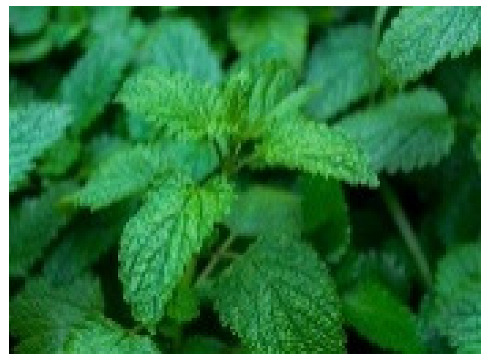	*Melissa officinalis* L.	Improved cardiac function; antioxidant properties (reduced AGEs); decreased BP and PWV; increased endothelium-dependent vascular relaxation.
	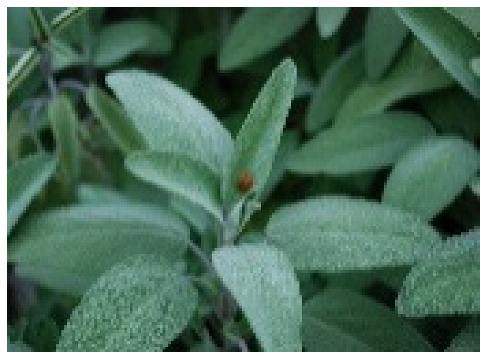	*Salvia miltiorrhiza* Bunge	Reduced PWV, inflammation, and oxidative stress; decreased glucose and cholesterol; increased insulin sensitivity.

PWV: Pulse Wave Velocity; BP: blood pressure; AGEs: Advanced Glycation End-products. The images of each plant are presented in the table and were obtained from the free picture website https://pixabay.com.

**Table 6:** Cardiovascular and metabolic effects of *Allium sativum* L.

Family	Name of the Plant		Effects of the Plant
Amaryllidaceae	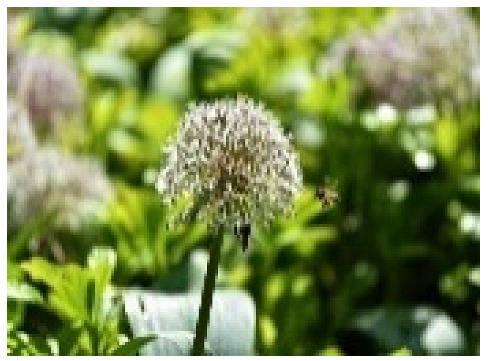	*Allium sativum* L.	Decreased PWV, BP, DBP, and AIx; AS reduction (reduced tunica media/adventitia/intima wall thickness); antihyperglycemic and antihypertensive activities; increased aortic elasticity; decreased blood TG.

PWV: Pulse Wave Velocity; BP: blood pressure; DBP: diastolic blood pressure; AIx: Augmentation Index; TG: triglycerides; AS: arterial stiffness. The image of *Allium sativum* L. introduced in the table was obtained from the free picture website https://pixabay.com.

**Table 7:** Cardiovascular and metabolic effects of *Camellia sinensis* L. (Kuntze).

Family	Name of the Plant		Effects of the Plant
Theaceae	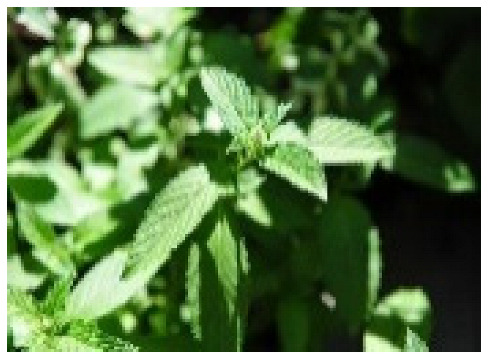	*Camellia sinensis* L. (Kuntze)	PWV decrease; oxidative stress and AS prevention; interaction with catecholamine signalling; endothelial function improvement; inhibition of extracellular matrix modifications.

PWV: Pulse Wave Velocity; AS: arterial stiffness. The image of *Camellia sinensis* L. (Kuntze) presented in the table was obtained from the free picture website https://pixabay.com.

**Table 8:** Cardiovascular and metabolic effects of *Carica papaya* L.

Family	Name of the Plant		Effects of the Plant
Caricaceae	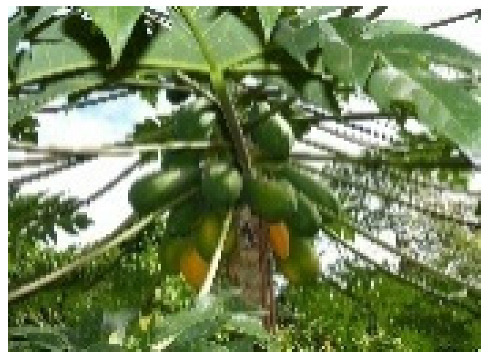	*Carica papaya* L.	Antihyperglycemic and antihypertensive effects; anti-ACE effects; stabilisation of BP; anti-inflammatory and antioxidant properties; increased NO production.

ACE: Angiotensin-Converting Enzyme; BP: blood pressure; NO: Nitric Oxide. The image of *Carica papaya* L. presented in the table was obtained from the free picture website https://pixabay.com.

**Table 9:** Cardiovascular and metabolic effects of *Citrullus lanatus* (Thunb.) Matsum. & Nakai.

Family	Name of the Plant		Effects of the Plant
Cucurbutaceae	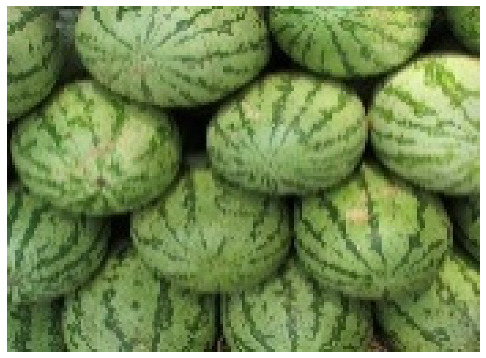	*Citrullus lanatus* (Thunb.) Matsum. & Nakai	Decrease in BP, AIx and PWV; vacular function improvement; increased NO production and vasodilation; enhanced antioxidant defence (SOD and GSH increased expressions).

BP: blood pressure; AIx: Augmentation Index; PWV: Pulse Wave Velocity; NO: Nitric Oxide; SOD: Superoxide Dismutase; GSH: Glutathione. The image of *Citrullus lanatus* (Thunb.) Matsum. & Nakai presented in the table was obtained from the free picture website https://pixabay.com.

**Table 10:** Cardiovascular and metabolic effects of *Curcuma longa* L.

Family	Name of the Plant		Effects of the Plant
Zingiberaceae	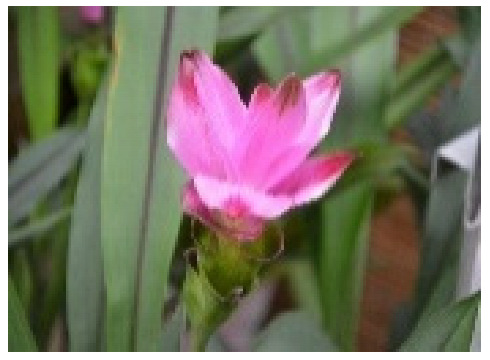	*Curcuma longa* L.	Decreased BP and PWV; increased eNOS-associated vasodilation and SOD2 expression; downregulation of NOX2 subunit p67^phox;^ reversed ageing-induced AS and increased aortic elasticity; free radical scavenging; decreases in MMP-2, MMP-9, and AGEs; reduced blood glucose and increased insulin sensitization.

BP: blood pressure; PWV: Pulse Wave Velocity; eNOS: endothelial Nitric Oxide Synthase; SOD2: Superoxide Dismutase 2; AS: Arterial Stiffness; MMP-2: Matrix Metalloproteinase 2; MMP-9: Matrix Metalloproteinase 9; AGEs: Advanced Glycation End-products. The image of *Curcuma longa* L. presented in the table was obtained from the free picture website https://pixabay.com.

**Table 11:** Cardiovascular and metabolic effects of *Garcinia mangostana* L.

Family	Name of the Plant		Effects of the Plant
Clusiaceae	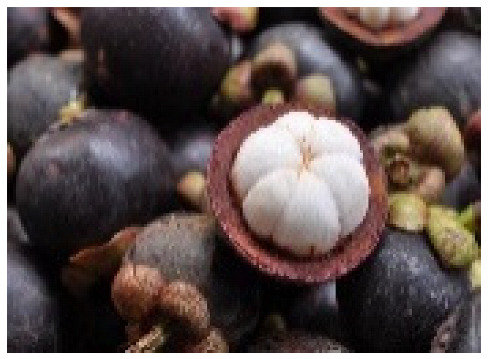	*Garcinia mangostana* L.	Reduced AS, AVI, and API; amplified aortic vasodilation; inhibition of vascular AGE formation; reduced Hb1Ac, total cholesterol, and LDL-C; reduced VCAM-1 expression; antioxidant and anti-inflammatory properties; inhibition of the ACE enzyme, RAAS, and SGLT-2.

AS: Arterial Stiffness; AVI: Arterial Velocity Pulse Index; API: Arterial Pressure Index; AGEs: Advanced Glycation End-products; Hb1Ac: glycated haemoglobin; LDL-C: Low-Density Lipoprotein Cholesterol; VCAM-1: Vascular Adhesion Molecule 1; ACE: Angiotensin Converting Enzyme; RAAS: Renin–Angiotensin–Aldosterone System; SGLT-2: Sodium-Glucose Cotransporter 2. The image of *Garcinia mangostana* L. presented in the table was obtained from the free picture website https://pixabay.com.

**Table 12:** Cardiovascular and metabolic effects of *Hylocereus undatus* (Haw.) Britton et Rose.

Family	Name of the Plant		Effects of the Plant
Cactaceae	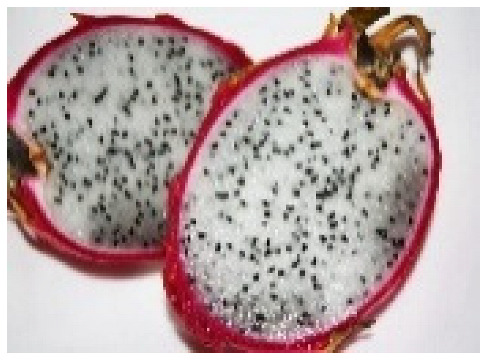	*Hylocereus undatus* (Haw.) Britton et Rose	Reduction in SBP, PWV, and AIx; MDA enzyme activity decrease and antioxidant defence amelioration (increased SOD and TAC); increased NO production and FMD; antidiabetic and antihypertensive properties (inhibition of SGLT-2 and/or GLP-1 RAs and/or DPP-4); inhibition of inflammatory cell proliferation/migration-induced aortic thickness.

SBP: systolic blood pressure; PWV: Pulse Wave Velocity; AIx: Augmentation Index; MDA: Malondialdehyde; SOD: Superoxide Dismutase; TAC: Total Antioxidant Capacity; NO: Nitric Oxide; FMD: Flow-Mediated Dilation; SGLT-2: Sodium-Glucose Cotransporter 2; GLP-1 RAs: Glucagon-Like Peptide 1 Receptor Agonists; DPP-4: Dipeptidyl Peptidase 4. The image of *Hylocereus undatus* (Haw.) Britton et Rose presented in the table was obtained from the free picture website https://pixabay.com.

**Table 13:** Cardiovascular and metabolic effects of *Labisia pumila* (Blume) Fern. -Vill. Var Alata.

Family	Name of the Plant		Effects of the Plant
Myrsinaceae	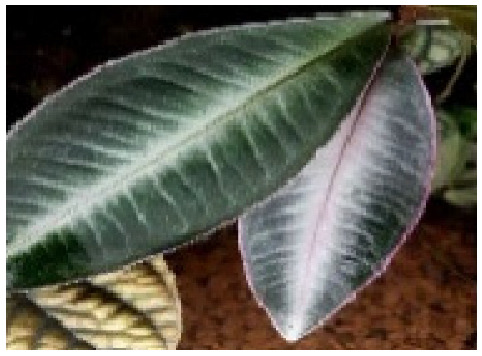	*Labisia pumila* (Blume) Fern. -Vill. Var Alata.	Prevention of AS-induced wall thickening; improvement of aortic elastin; increased glucose uptake and insulin sensitivity (upregulation of PPAR γ); increased NO and EDHF-dependent vasorelaxation.

AS: Arterial Stiffness; PPARγ: Peroxisome Proliferator Activated Receptor; NO: Nitric Oxide; EDHF: Endothelium-Derived Hyperpolarizing Factor. The image of *Labisia pumila* (Blume) Fern. -Vill. Var Alata introduced in the table was obtained from the free picture website https://pixabay.com.

**Table 14:** Cardiovascular and metabolic effects of *Phaleria macrocarpa* (Scheff.) Boerl.

Family	Name of the Plant		Effects of the Plant
Thymelaeaceae	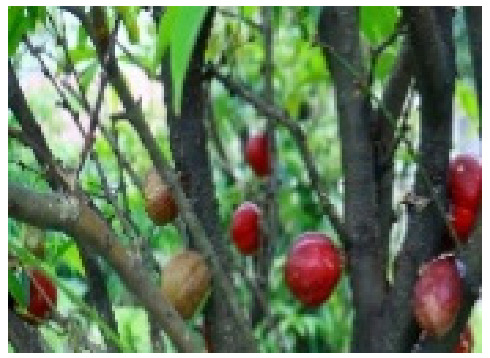	*Phaleria macrocarpa* (Scheff.) Boerl.	Reduced glycemia, BP, PWV, blood cholesterol, and insulin resistance; increased aorta endothelial-dependent relaxation; AS reduction.

BP: blood pressure; PWV: Pulse Wave Velocity; AS: Arterial Stiffness. The image of *Phaleria macrocarpa* (Scheff.) Boerl. presented in the table was obtained from the free picture website https://pixabay.com.

**Table 15:** Cardiovascular and metabolic effects of *Phyllantus emblica* L.

Family	Name of the Plant		Effects of the Plant
Phyllanthaceae	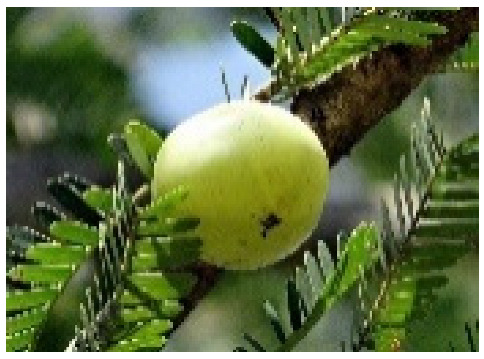	*Phyllantus emblica* L.	Increased endothelial NO bioavailability and vasorelaxation; decreased MDA enzyme levels; decreased tunica intima layer thickness and restored elastin structure; antioxidant effect; decreased BP, AIx, PWV, RI, blood glucose, urine sugar levels, and cholesterol; increased insulin sensitisation and haemoglobin plasma concentration; limitation of vascular inflammation progression and VSMC growth and migration.

NO: Nitric Oxide; MDA: Malondialdehyde; BP: blood pressure; AIx: Augmentation Index; PWV: Pulse Wave Velocity; RI: Reflexion Index; VSMC: Vascular Smooth Muscle Cell. The image of *Phyllantus emblica* L. presented in the table was obtained from the free picture website https://pixabay.com.

**Table 16:** Cardiovascular and metabolic effects of *Moringa oleifera* Lam.

Family	Name of the Plant		Effects of the Plant
Moringaceae	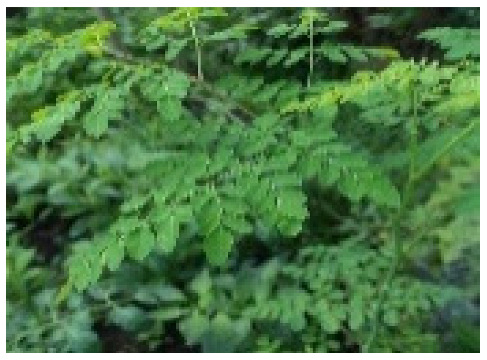	*Moringa oleifera* Lam.	Reduced MAP, improvement of endothelium-dependent vasodilation, eNOS function, and endothelial NO production; Akt signalling increase; Arginase-1 downregulation; increased SIRT1 expression and activity; anti-inflammatory and antioxidant vascular effects.

MAP: Mean Arterial Pressure; eNOS: endothelial Nitric Oxide synthase; NO: Nitric Oxide; SIRT1: Sirtuin 1. The image of *Moringa oleifera* Lam. presented in the table was obtained from the free picture website https://pixabay.com.

## Data Availability

No new data were created or analysed in this study. Data sharing is not applicable to this article.

## Abbreviations

The following abbreviations are used in this manuscript:

ACE	Angiotensin-Converting Enzyme
ACh	Acetylcholine
AGEs	Advanced Glycation End-products
AIx	Augmentation Index
AMPK	Adenosine Monophosphate–Activated Protein Kinase
Ang II	Angiotensin II
AP-1	Activator Protein 1
API	Arterial Pressure Index
ARA II	Angiotensin II Receptors
ARBs	Angiotensin Receptor Blockers
AS	Arterial Stiffness
AT1	Angiotensin II Receptor Type 1
AVI	Arterial Velocity Pulse Index
ba-PWV	Brachial–Ankle Pulse Wave Velocity
BBs	Beta-Blockers
BP	Blood pressure
CAVI	Cardio–Ankle Vascular Index
CCBs	Calcium Channel Blockers
cf-PWV	Carotid–Femoral Pulse Wave Velocity
cGMP	Cyclic Guanosine Monophosphate
CKD	Chronic kidney disease
CRP	C-reactive protein
Cu/Zn-SOD	Copper/zinc-containing Superoxide Dismutase
DBP	Diastolic Blood Pressure
DPP4i	Dipeptidyl Peptidase 4 Inhibitors
DRI	Direct Renin Inhibitors
EDHFs	Endothelium-Derived Hyperpolarising Factors
Einc	Incremental Elastic Modulus
Ep	Elastic modulus
ERK	Extracellular Signal-Regulated Kinases
ET-1	Endothelin 1
EVR	Elastic Vascular Resistance
FMD	Flow-Mediated Dilation
GLP-1 RA	Glucagon-Like Peptide 1 Receptor Agonist
GLUT	Glucose Transporter
GSH	Glutathione (reduced)
HDL	High-Density Lipoprotein
HFD	High-fat diet
HFFD	High-Fat-high-Fructose Diet
HMG-CoA	3-Hydroxy-3-Methyl-Glutaryl-Coenzyme A
H_2_O_2_	Hydrogen Peroxide
hsCRP	High-sensitive inflammatory marker C-Reactive Protein
HSD	High-salt diet
HUVECs	Human Umbilical Vein Endothelial Cells
ICAM-1	Intercellular Adhesion Molecule 1
IFNγ	Interferon Gamma
IL-1ß	Interleukin 1 Beta
IL-6	Interleukin 6
iNOS	Inducible Nitric Oxide Synthase
IRS-1	Insulin Receptor Substrate 1
KRG	Korean Red Ginseng
LB	Lemon Balm
LDH	Lactate Dehydrogenase
LDL-C	Low-Density Lipoprotein Cholesterol
L-NAME	N-Nitro-L-Arginine Methyl Ester hydrochloride
LOXL-1	Lysyl-Oxidase-Like 1
MAP	Mean Arterial Pressure
MCP-1	Monocyte Chemotactic Protein 1
MDA	Malondialdehyde
MF	Metformin
MMP	Matrix Metalloproteinase
Mn-SOD	Manganese Superoxide Dismutase
NaCl	Sodium Chloride
NADPH	Nicotinamide Adenine Dinucleotide Phosphate
NF-κB	Nuclear Factor Kappa-B
NO	Nitric Oxide
eNOS	endothelial Nitric Oxide Synthase
NOX1	NADPH oxidase 1
NOX2	NADPH oxidase 2
NOX4	NADPH oxidase 4
Nrf2	Nuclear Factor E2-related Factor 2
OPG	OsteoProteGerin
OVX	Ovariectomized
PDGF	Platelet-derived growth factor
PI_3_K	Phosphoinositide 3-Kinase
PKA	Protein Kinase A
PKB	Protein Kinase B
PPAR	Peroxisome Proliferator Activated Receptor
PP	Central Pulse Pressure
PWT	Pulse Wave Transmission
RA	Rosmarinic Acid
Rac1	Ras-related C3 botulinum toxine Substrate 1
RAGE	AGEs receptors
RAAS	Renin–Angiotensin–Aldosterone System
RhoA	Ras Homolog family member A
RI	Reflexion Index
ROCK	Rho-associated Kinases
ROS	Reactive Oxygen Species
SBP	Systolic Blood Pressure
SD	Sprague–Dawley
SGLT-2	Sodium-Glucose Cotransporter 2
SHR	Spontaneously Hypertensive Rats
SIRT1	Sirtuin 1
Smad	Suppressor Mothers Against Decapentaplegic
SOD	Superoxide Dismutase
STZ	Streptozotocin
TAG	Triacylglycerol
TBARs	Thiobarbituric Acid Reactive substance
TC	Total Cholesterol
TE	Tropoelastin
TG	Triglycerides
TGF-ß1	Transforming Growth Factor Beta 1
THC	Tetrahydrocurcumin
TNF-α	Tumour Necrosis Factor-Alpha
VCAM-1	Vascular Adhesion Molecule 1
VMSC	Vascular Smooth Muscle Cell
WC	Waist circumference
WKY	Wistar Kyoto
ω-3	Omega-3
